# Comparison of Two Chelator Scaffolds as Basis for Cholecystokinin-2 Receptor Targeting Bimodal Imaging Probes

**DOI:** 10.3390/ph17121569

**Published:** 2024-11-22

**Authors:** Giacomo Gariglio, Katerina Bendova, Martin Hermann, Asta Olafsdottir, Jane K. Sosabowski, Milos Petrik, Elisabeth von Guggenberg, Clemens Decristoforo

**Affiliations:** 1 Department of Nuclear Medicine, Medical University of Innsbruck, 6020 Innsbruck, Austria; 2 Institute of Molecular and Translational Medicine, Faculty of Medicine and Dentistry, Palacky University, 77900 Olomouc, Czech Republic; 3 Department of Anesthesiology and Critical Care Medicine, Medical University Innsbruck, 6020 Innsbruck, Austria; 4 Perceptive Discovery, Hammersmith Hospital, Imperial College London, London W12 0NN, UK; 5 Centre for Cancer Biomarkers and Biotherapeutics, Barts Cancer Institute, Queen Mary University of London, London E1 4NS, UK; 6 Czech Advanced Technology and Research Institute, Palacky University, 77900 Olomouc, Czech Republic; 7 Laboratory of Experimental Medicine, University Hospital, 77900 Olomouc, Czech Republic

**Keywords:** dual-modality imaging agent, PET, fluorescence guided surgery, cholecystokinin-2 receptor, gallium-68, TRAP, FSC, SulfoCy5.5

## Abstract

**Background/Objectives**: Dual-modality probes, combining positron emission tomography (PET) with fluorescence imaging (FI) capabilities in a single molecule, are of high relevance for the accurate staging and guided resection of tumours. We herein present a pair of candidates targeting the cholecystokinin-2 receptor (CCK2R), namely [^68^Ga]Ga-CyTMG and [^68^Ga]Ga-CyFMG. In these probes, the SulfoCy5.5 fluorophore and two units of a CCK2R-binding motif are coupled to the chelator acting as a core scaffold, triazacyclononane-phosphinic acid (TRAP), and Fusarinine C (FSC), respectively. Using this approach, we investigated the influence of these chelators on the final properties. **Methods**: The synthetic strategy to both precursors was based on the stoichiometric conjugation of the components via click chemistry. The characterization in vitro included the evaluation of the CCK2R affinity and internalization in A431-CCK2R cells. Ex vivo biodistribution as well as PET and FI studies were performed in xenografted mice. **Results**: ^68^Ga labelling was accomplished with high radiochemical yield and purity for both precursors. A CCK2R affinity in the subnanomolar range of the conjugates and a receptor-specific uptake of the radioligands in cells were observed. In A431-CCK2R/A431-mock xenografted mice, the investigated compounds showed specific accumulation in the tumours and reduced off-target uptake compared to a previously developed compound. Higher accumulation and prolonged retention in the kidneys were observed for [^68^Ga]Ga-CyTMG when compared to [^68^Ga]Ga-CyFMG. **Conclusions:** Despite the promising targeting properties observed, further probe optimization is required to achieve enhanced imaging contrast at early timepoints. Additionally, the results indicate a distinct influence of the chelators in terms of renal accumulation and retention.

## 1. Introduction

As estimated by the World Health Organization (WHO) in 2019, cancer remains the primary or secondary cause of mortality before the age of 70 years in over 60% of countries globally, and its incidence is expected to increase over the next 50 years [1,2]. For an individual diagnosed with cancer, delayed diagnosis and incomplete treatment may result in poor prognosis and increased mortality. Consequently, an early detection of primary lesions and metastases, in addition to effective surgical intervention, can significantly improve patient outcomes [3].

In recent decades, molecular imaging, which includes a range of techniques such as magnetic resonance imaging (MRI), optical and near-infrared fluorescence imaging (FI), ultrasound imaging (USI), and nuclear medicine imaging techniques, including positron emission tomography (PET) and single-photon emission computed tomography (SPECT), has attained a crucial role in the field of cancer diagnostics and therapeutics [4].

In order to fully exploit their distinctive and complementary strengths, PET and FI can be employed in a synergistic approach to visualize tumour lesions pre- and intra-operatively, respectively. PET offers detection sensitivity at the subnanomolar level without penetration-related limitations in living tissues. A single radiotracer injection provides non-invasive, functional, and quantitative whole-body data.

Fluorescence imaging in the near-infrared region of 650–900 nm is well suited to surgical guidance, as it enables a real-time visualization of tumour margins with a high spatial resolution (down to tens of nanometers) and a sensitivity of detection in the nanomolar range. This enables the complete and minimally invasive resection of cancerous tissue [5]. Nevertheless, the low penetration of the fluorescent signal precludes quantitative information and whole-body observation. So far, cyanine dyes have been successfully employed for fluorescence-guided surgery. Recently, OTL38, a folate receptor-targeted FI agent featuring an indocyanine green-like near-infrared dye, has gained FDA approval. This achievement substantiates the clinical utility of this approach [6,7].

Given the comparable sensitivity and complementary information provided, nuclear and fluorescence imaging capabilities can be combined in a single bimodal PET/FI agent. This approach ensures the optimal correlation of the radioactive and fluorescent signals, thereby also facilitating cross-validation of the two techniques. A bimodal PET/FI agent can therefore be used to enable preoperative nuclear imaging for surgical planning and intraoperative real-time guidance aiming to complete tumour resection, ideally via a single probe injection [8,9,10].

Over the last twenty years, CCK2R, expressed at high incidence and density in medullary thyroid carcinoma (MTC) and small-cell lung cancer (SCLC) among other malignancies, emerged as an attractive target for the development of diagnostic and therapeutic targeted radiopharmaceuticals based on gastrin and cholecystokinin analogues [11,12]. In the specific context of MTC, local lymph node metastases are prevalent and frequently undetected during preoperative neck ultrasonography [13,14,15]. Consequently, despite total thyroidectomy and central lymph node dissection, approximately 50% of patients exhibit persistent disease or develop recurrent lesions [16,17].

To enable complete resection, fluorescent imaging agents based on peptide agonists and small-molecule antagonists for the intraoperative surgical guidance of MTC have been investigated [18,19]. Our group reported the first dimeric bimodal PET/FI agent targeting the CCK2R, [^68^Ga]Ga-Sulfo-Cy7-FSC-MG [20]. In this proof-of-principle study, the Fusarinine C (FSC) chelator was used as a central scaffold and was conjugated to a near-infrared fluorophore (Sulfo-Cyanine7) and to two units of a minigastrin analogue (MG11). *In vivo*, this dimeric bimodal probe demonstrated specific but low tumour uptake accompanied by slow blood clearance and retention in non-targeted tissues, resulting in suboptimal imaging contrast at early timepoints.

In this study, we aimed to improve this initial approach and investigated the properties of a pair of novel candidates based on a more hydrophilic fluorophore (SulfoCy5.5) and using two units of a modified CCK2R-binding motif with reduced susceptibility to enzymatic degradation. These probes, designated CyTMG and CyFMG, differ exclusively for the multifunctional chelator used as the core scaffold, respectively, TRAP and FSC. In vitro characterization included internalization experiments with A431 cells stably transfected with human CCK2R (A431-CCK2R) and mock-transfected cells (A431-mock), as well as competitive binding assays. Subsequently, we validated the in vivo targeting ability using tumour xenografted mice. In addition, by performing imaging studies, we assessed the potential for preoperative lesion detection and intraoperative tumour margin delineation.

## 2. Results

### 2.1. Precursor Preparation

The siderophore FSC was extracted from *Aspergillus fumigatus* culture and iron salt was added to obtain the corresponding metal complex. This step is necessary to prevent the involvement of the hydroxamates in any side reactions during the synthesis. The stabilized CCK2R-binding motif Trp-(N-Me)Nle-Asp-1-Nal-NH_2_ derived from DOTA-MGS5 and its conjugation to the PEG4-Azide linker were obtained through solid-phase peptide synthesis [21].

**Figure 1:**
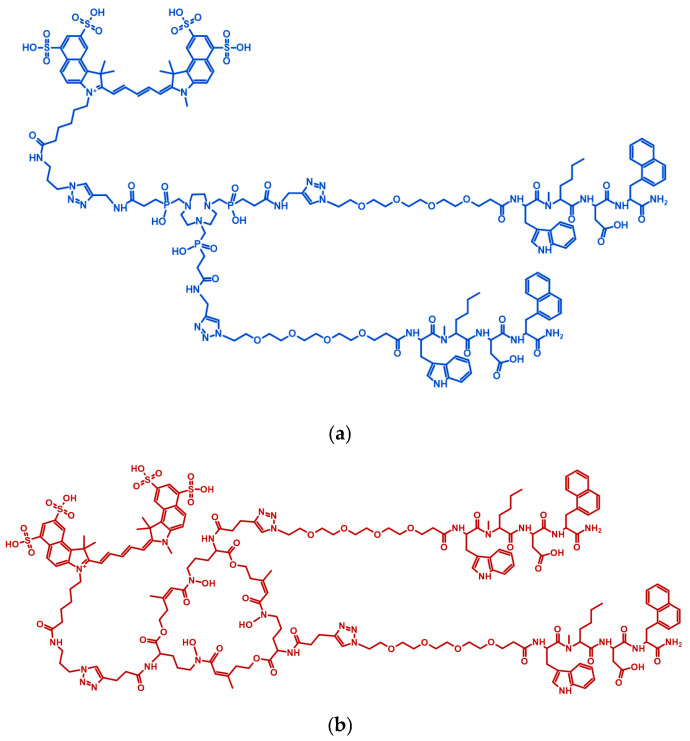
Chemical structure of conjugates. Structure of CyTMG (**a**) and CyFMG (**b**).

The synthetic strategy to both labelling precursors started with the derivatization of the chelator via classic amide coupling to introduce alkyne functionalities (Figure S1). The three carboxylic acids of TRAP were functionalized with propargylamine, while the three amino groups of [Fe]FSC were derivatized with 4-pentynoic acid. Next, the SulfoCy5.5 fluorophore and two units of the pegylated CCK2R-binding motif were subsequently introduced by a stoichiometric Cu(I)-catalyzed azide–alkyne cycloaddition (CuAAC) click reaction. Eventually, the coordinated metal in [Cu]CyTMG and [Fe]CyFMG was removed with an excess of EDTA as a competing chelator to provide the labelling precursors in moderate yield (respectively, 33 and 23%) and sufficient chemical purity (>95%) (Figure 1).

### 2.2. Radiolabelling with Gallium-68

Both CyTMG and CyFMG were radiolabelled within minutes with gallium-68 in high radiochemical purity (>98%), as confirmed by RP-HPLC and radio-iTLC (Figures S18–S21), and at elevated molar activity (up to 30 GBq/µmol non-optimized). With these labelling performances, no further purification was necessary, and the radiolabelled tracers were directly used for all experiments.

### 2.3. In Vitro Characterization

Distribution coefficients (*LogD*_pH7.4_), protein binding, and stability evaluations in human serum and phosphate-buffered saline (PBS, pH 7.4) for the two hybrid imaging agents are summarized in Table 1. [^68^Ga]Ga-CyTMG and [^68^Ga]Ga-CyFMG showed *LogD*_pH7.4_ values of −1.78 ± 0.16 and −1.53 ± 0.10 in agreement with the radio-RP-HPLC retention time observed (15.5 and 16.1 min, respectively). The stability was assessed in PBS and human serum for 1, 2, and 4 h after incubation by radio-RP-HPLC (Table 1, Figure S22). Over the timepoints investigated, both radiocompounds displayed high stability in human serum with less than 4% of degradation or radionuclide release, while binding to serum proteins was in the range of 50–70%.

**Figure 2:**
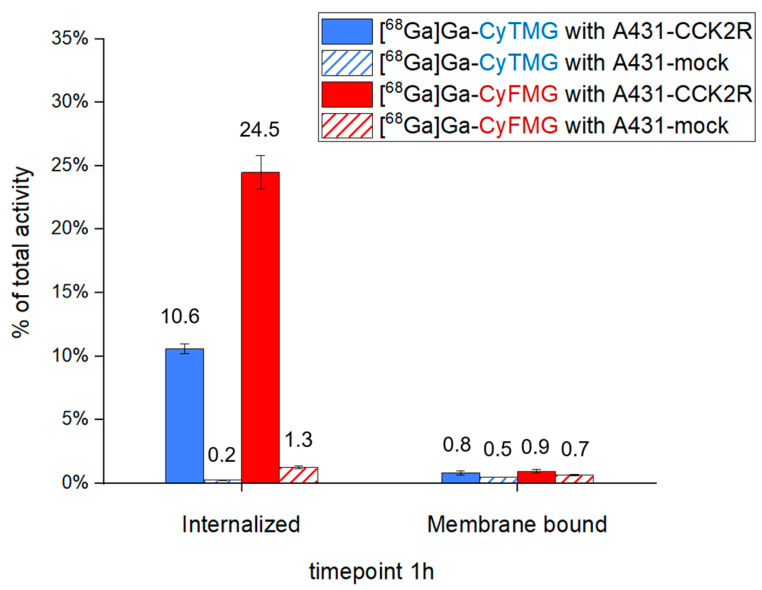
Cell-associated radioactivity determined for [^68^Ga]Ga-CyTMG and [^68^Ga]Ga-CyFMG in A431-CCK2R and A431-mock cells. The values are reported as means of three independent experiments.

**Figure 3:**
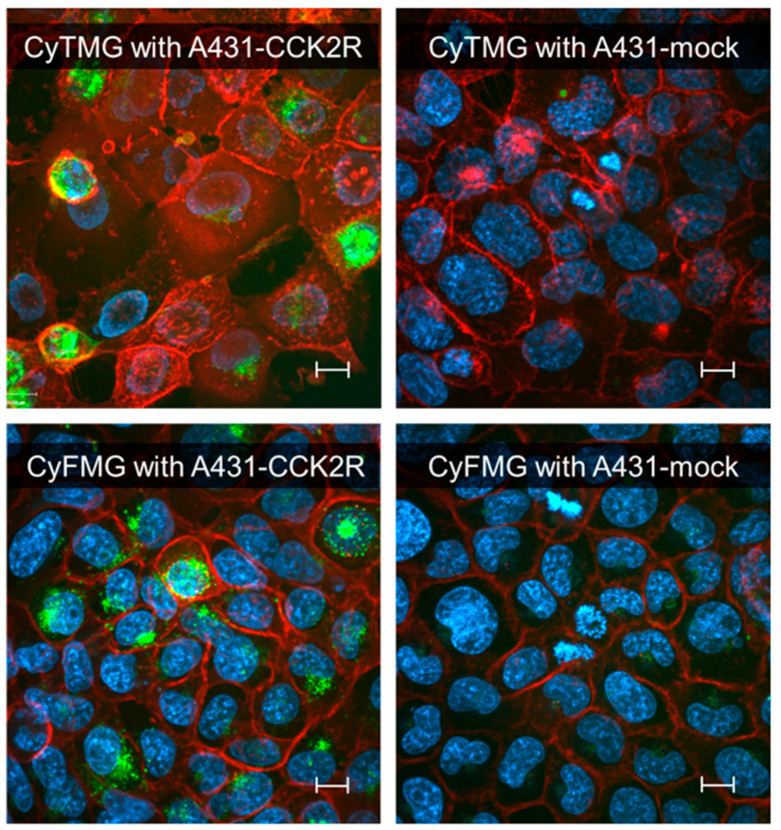
Cell-associated fluorescence determined for CyTMG and CyFMG using A431-CCK2R and A431-mock cells. The images reported overlay the SulfoCy5.5 channel (green) showing the fluorescent agent, the WGA-Alexa fluor channel (red) showing the cell membrane, and the HOECHST channel (blue) showing the nucleus. Magnification is equal for all images (scale bar: 16 µm).

**Figure 4:**
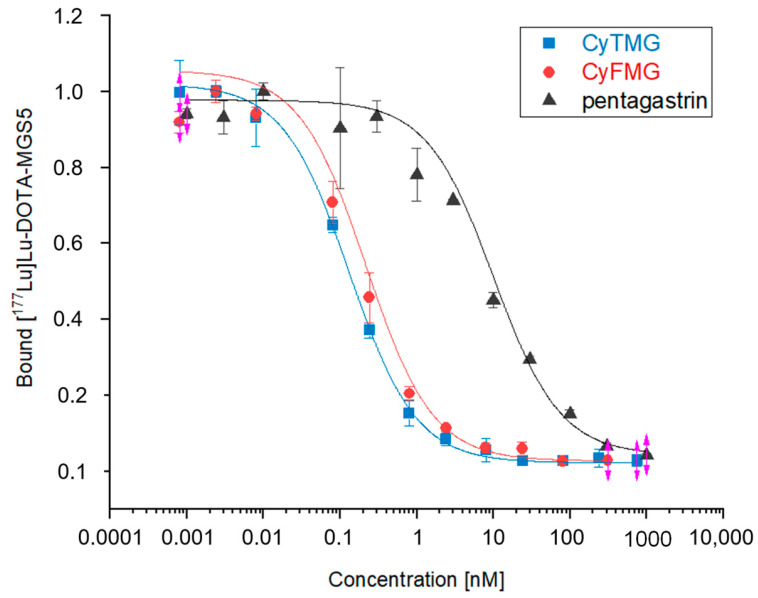
Receptor-binding affinity of CyTMG and CyFMG on A431-CCK2R cells. IC50 values are reported as means of two independent experiments, each performed in triplicate.

A head-to-head comparison of the cell-associated radioactivity was performed in cell uptake studies with A431-CCK2R and A431-mock cells (Figure 2). For both radiotracers, the results showed cellular uptake of radioactivity mediated by CCK2R after 1 h of incubation. Levels of 10.59 ± 0.40% and 24.48 ± 1.33% for the internalized fraction were found for [^68^Ga]Ga-CyTMG and [^68^Ga]Ga-CyFMG, respectively, along with negligible radioactivity found in the membrane fraction. Confocal fluorescence microscopy experiments confirmed these findings. The fluorescent signal associated with the bimodal compounds was located close to the nucleus of the A431-CCK2R cells, confirming the CCK2R-mediated intracellular uptake of the compounds, while no detectable signal was observed in the control cells incubated with the radiocompounds (Figure 3). Elevated CCK2R affinity was observed for both metal-free precursors. CyTMG showed an IC_50_ value of 0.24 ± 0.16 nM, while CyFMG showed a value of 0.37 ± 0.22 nM. For the pentagastrin reference, the IC_50_ was found in the nanomolar range (8.66 ± 1.55 nM) (Figure 4).

### 2.4. In Vivo Characterization

**Figure 5:**
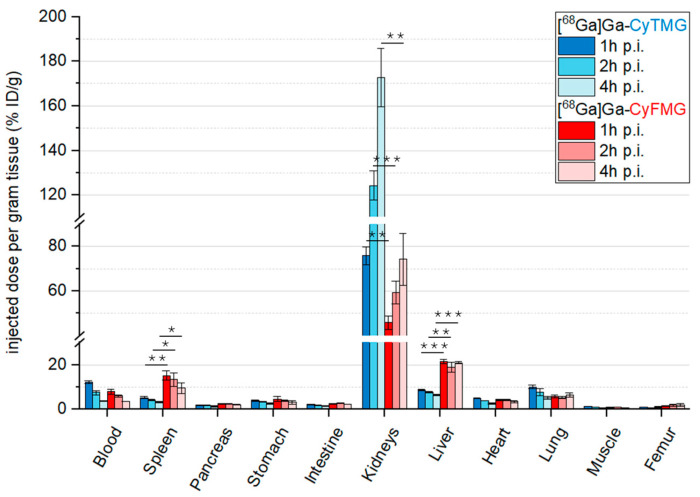
Ex vivo biodistribution studies in healthy BALB/C mice (n = 3) performed at 1, 2, and 4 h p.i. for [^68^Ga]Ga-CyTMG and for [^68^Ga]Ga-CyFMG (amount injected: 0.14 nmol, 1.5 MBq). The asterisks represent the level of significance determined by using the *p* value (*: 0.01 < *p* < 0.05; **: 0.001 < *p* < 0.01; ***: *p* < 0.001).

The results of the ex vivo biodistribution studies of the two radiotracers on healthy mice are reported in Figure 5.

**Figure 6:**
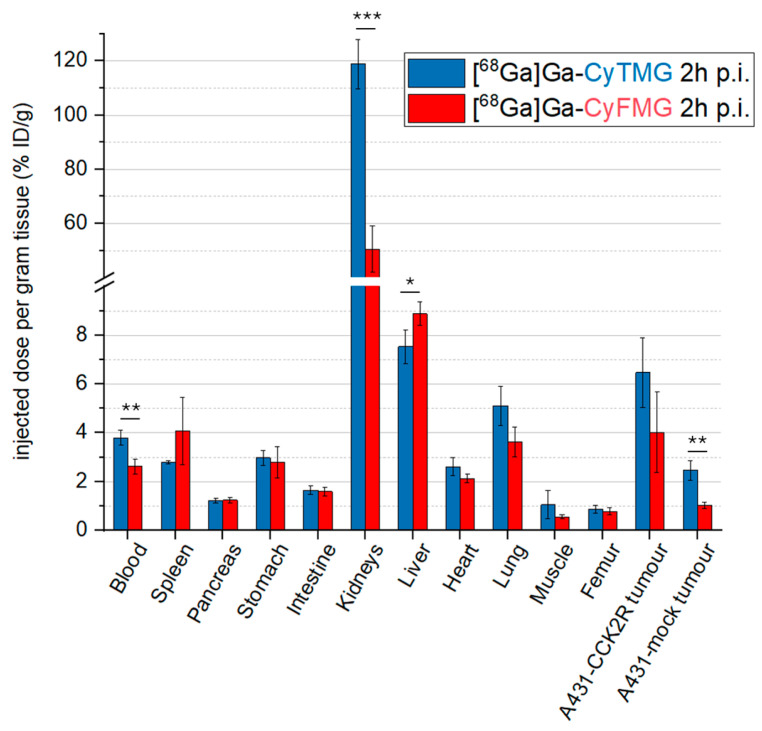
Ex vivo biodistribution studies in A431-CCK2R and A431-mock xenografted mice. BALB/C mice (n = 3) performed for 2 h p.i. for [^68^Ga]Ga-CyTMG and [^68^Ga]Ga-CyFMG (amount injected: 0.05 nmol, 1.0 MBq). The asterisks represent the level of significance determined by using the *p* value (*: 0.01 < *p* < 0.05; **: 0.001 < *p* < 0.01; ***: *p* < 0.001).

**Figure 7:**
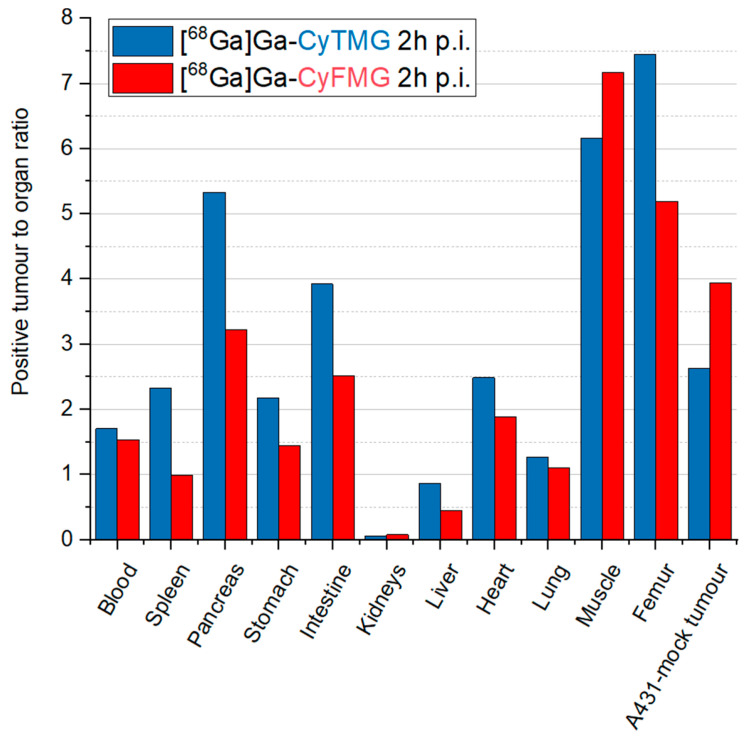
Tumour-to-organ ratios for A431 xenografts of mice injected with [^68^Ga]Ga-CyTMG and [^68^Ga]Ga-CyFMG.

Slow pharmacokinetics were found, as indicated by the relatively high radioactivity levels still present in the blood at 4 h p.i. (~3.60% ID/g). This correlates with the uptake values in other highly perfused organs such as the heart, the lungs and the liver. When comparing the organ uptake of the two compounds, [^68^Ga]Ga-CyFMG demonstrated approximately threefold higher accumulation in the spleen and liver at all timepoints. Both radiotracers showed pronounced and increasing uptake values in the kidneys during the time interval investigated. The accumulation of radioactivity in the kidney was approximately two times higher for [^68^Ga]Ga-CyTMG in comparison to [^68^Ga]Ga-CyFMG (% ID/g for [^68^Ga]Ga-CyTMG and [^68^Ga]Ga-CyFMG, with values of 75.81 ± 3.88% vs. 45.86 ± 3.03% at 1 h, 124.31 ± 6.46 vs. 59.28 ± 5.04% at 2 h, and 172.64 ± 13.04 vs. 74.29 ± 11.67% at 4 h). Ex vivo biodistribution at 2 h p.i. in A431-CCK2R and A431-mock xenografted mice are reported in Figure 6 and the corresponding tumour-to-organ ratios are shown in Figure 7. The accumulation of radioactivity in the A431-CCK2R xenografts was found to be 6.47 ± 1.47% and 4.02 ± 1.64% ID/g for [^68^Ga]Ga-CyTMG and [^68^Ga]Ga-CyFMG, respectively, with significantly lower values in A431-mock tumours (2.46 ± 0.40% and 1.02 ± 0.13% ID/g). The ratio of CCK2R-positive tumours and A431-mock xenografts was 2.63 and 3.94, respectively. When comparing the overall biodistribution profile of the two radiotracers, the major difference observed was the kidney uptake with values of 118.89 ± 9.12% found for [^68^Ga]Ga-CyTMG, whereas this was significantly reduced to values of 50.66 ± 8.42% (*p* = 0.0003) for [^68^Ga]Ga-CyFMG only. Only the uptake in the liver was higher in mice injected with [^68^Ga]Ga-CyFMG. These trends were consistent with what was observed in healthy BALB/c mice. With the exception of the A431-mock xenograft and the muscle, higher tumour-to-organ ratios were found for [^68^Ga]Ga-CyTMG versus [^68^Ga]Ga-CyFMG. Compared to the results obtained in the healthy animals, lower levels in the blood, in highly perfused organs, and in the spleen were found for both radiotracers in xenografted BALB/c nude mice. In addition, somewhat lower uptakes in the stomach, intestine and in the liver were observed for [^68^Ga]Ga-CyFMG.

**Figure 8:**
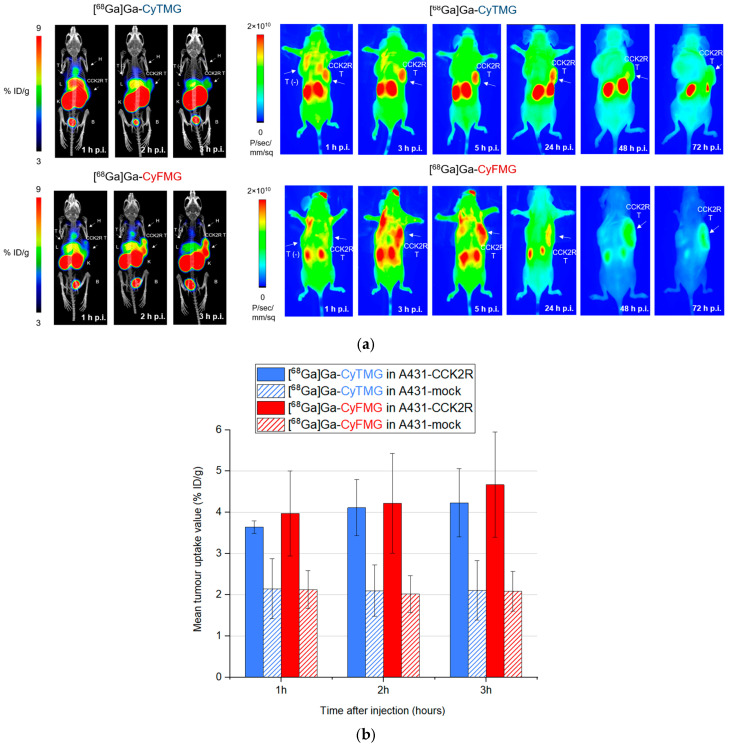
(**a**) Imaging study of two A431-CCK2R and A431-mock xenografted mice injected with [^68^Ga]Ga-CyTMG or [^68^Ga]Ga-CyFMG (amount injected: 0.3 nmol, 6.0 MBq). Static PET/CT MIP images (left) and corresponding near-infrared fluorescence images (right) at various timepoints. B: bladder; H: heart; L: liver; T: tumour; K: kidneys. (**b**) Comparison of uptake in A431-CCK2R and A431-mock tumours of animals injected with [^68^Ga]Ga-CyTMG or [^68^Ga]Ga-CyFMG. Values are calculated in manually drawn ROIs fitting to the tumours. Results are expressed as mean uptake values (n = 3).

Animal imaging studies, consisting of both PET/CT and fluorescence imaging, were performed in each mouse studied. The PET/CT images obtained with the two radiotracers confirmed the results from the ex vivo biodistribution profile, demonstrating an elevated accumulation of radioactivity in the kidneys, together with moderate uptake in the liver and in the heart (Figure 8a). Receptor-specific uptake could be clearly visualized in A431-CCK2R xenografts, while no uptake was visible in A431-mock tumours. Quantitative analysis of the images confirmed a good tumour retention of [^68^Ga]Ga-CyTMG and [^68^Ga]Ga-CyFMG, with uptake values slightly increasing over time. (Figure 8b). Due to the decreasing signal in non-targeted tissue, the best contrast for PET/CT images was achieved at 3 h after injection. The A431-CCK2R xenografts were clearly visualized also by fluorescence imaging (Figure 8a). For [^68^Ga]Ga-CyTMG, the cancerous tissue could be well delineated starting from 1 h after injection, while in the case of [^68^Ga]Ga-CyFMG, this was possible starting from 3 h after injection. The best target-to-background contrast was reached from 3 h to 24 h and from 5 h to 72 h after administration, respectively, for [^68^Ga]Ga-CyTMG and [^68^Ga]Ga-CyFMG. A noteworthy observation derived from these results is that in the case of [^68^Ga]Ga-CyTMG, the signal in the kidneys persisted for up to 72 h post-injection. In contrast, in the case of [^68^Ga]Ga-CyFMG, this uptake visibly declined from 24 h to disappear completely at 72 h after injection.

**Figure 9:**
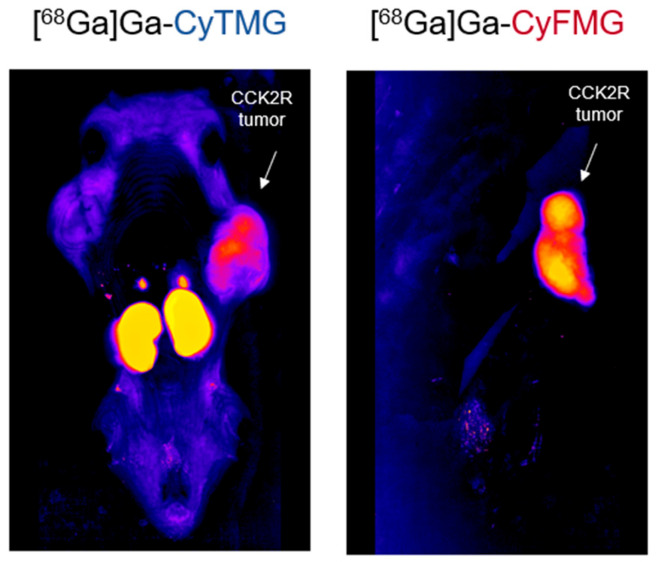
Cryo-fluorescence tomography of two A431-CCK2R and A431-mock xenografted mice injected with [^68^Ga]Ga-CyTMG or [^68^Ga]Ga-CyFMG performed for 72 h p.i.

Cryo-fluorescence tomography imaging confirmed the excellent targeting properties to visualize the CCK2R tumour at the latest timepoint of 72 h p.i. and demonstrated the absence of non-targeted fluorescence other than the renal retention observed for [^68^Ga]Ga-CyTMG (Figure 9).

## 3. Discussion

As bimodal PET/FI probes have great potential in improving the surgical outcomes of patients affected by MTC, we initially developed [^68^Ga]Ga-Sulfo-Cy7-FSC-MG, the first example of a CCK2R-targeting dual-modality imaging agent, which showed encouraging results in terms of receptor affinity, tumour accumulation, and tumour retention [20]. These favourable properties can be attributed to the dimerization of the minigastrin-11 (MG11) peptide, as multivalent probes are capable of binding simultaneously to multiple receptors and have a higher chance of rebinding to the target in their original intact form or even as metabolites [22,23,24,25]. On the other hand, the slow blood clearance and non-specific accumulation in the spleen, liver, and kidneys demonstrated by [^68^Ga]Ga-Sulfo-Cy7-FSC-MG indicated an opportunity for improvement [20]. These properties relate both to the considered moieties (fluorophore, targeting unit, and chelator) and to their specific combination. In this study, we maintained the overall structural configuration of Sulfo-Cy7-FSC-MG but thoughtfully varied each domain to improve this initial concept to move one step closer to clinical application.

The suboptimal properties of [^68^Ga]Ga-Sulfo-Cy7-FSC-MG can be related primarily to the introduction of the Sulfo-Cyanine7 fluorophore. Compared to the compound lacking the fluorophore, [^68^Ga]Ga-mAcFSC-(mal-MG11)_2_, the bimodal probe showed lower hydrophilicity, a distinctively higher protein binding (4.5-fold), and inferior CCK2R affinity [20,25]. Preclinical and clinical studies demonstrated that far-red pentamethine cyanine dyes (650 nm ≤ λ_em_ ≤ 700 nm) also support fluorescence-guided surgery and compared to their heptamethine counterparts, their use has the advantage of being compatible with standard confocal fluorescence microscopes. In a systematic study of the influence of the length of the polymethine chain, a cyanine 5-based bimodal tracer was reported to exhibit not only higher photophysical properties but also lower lipophilicity, protein binding, and non-specific tissue uptake when compared to the cyanine 7 derivative [26]. In light of these considerations, we selected the hydrophilic tetrasulfonated SulfoCy5.5 as the fluorescent tag for the new conjugates developed.

The MG11 targeting sequence used in Sulfo-Cy7-FSC-MG was reported to be sensitive to enzymatic degradation, leading to cleavage within the C-terminal receptor-specific sequence Tyr-Gly-Trp-Met-Asp-Phe-NH_2_ [22,27]. To address this issue, in this study, we used the shorter and chemically stabilized CCK2R-binding motif Trp-(N-Me)Nle-Asp-1NaI-NH_2_ of DOTA-MGS5, a minigastrin derivative that was recently developed by our group. DOTA-MGS5 labelled with different radiometals showed excellent metabolic stability, leading to highly enhanced tumour targeting [21]. To counter the lipophilicity related to the introduction of the bulky amino acids, we decided to use the hydrophilic PEG4 linkers as a spacer between the chelator and the targeting units.

As an alternative multifunctional chelator to Fusarinine C (FSC), we considered triazacyclononane-phosphinic acid (TRAP) [28,29]. In addition to its remarkable Gallium-68 labelling properties, this ligand can also be derivatized on three available carboxylic groups without compromising the integrity of the complexation site, allowing for the synthesis of innovative tracers, including dimeric dual-modality imaging agents. Unlike FSC, this chelator is commercially available and has already been tested in humans [30,31].

On the basis of TRAP and of the above-mentioned domains, we prepared CyTMG, a novel dual-modality imaging agent targeting CCK2R. To investigate the influence of the chelator moiety on the behaviour of the final compound, we also synthesized CyFMG, an analogous compound based on FSC.

Both dual-modality imaging agents showed moderate hydrophilic properties. In particular, [^68^Ga]Ga-CyTMG was found to be slightly more hydrophilic than [^68^Ga]Ga-CyFMG according to both *LogD*_pH7.4_ and radio-HPLC retention times. This result can be explained by considering the smaller molecular size and presence of the highly polar phosphinic groups of the TRAP chelator. By comparing the *LogD*_pH7.4_ of the fluorescent diacetyl(FSC) derivative [^68^Ga]Ga-SulfoCy7-DAFC (*LogD*_pH7.4_ = −2.19 ± 0.07) [32] with the one of Sulfo-Cy7-FSC-MG (*LogD*_pH7.4_ = −1.90 ± 0.17) and of [^68^Ga]Ga-CyFMG (*LogD*_pH7.4_ = −1.53 ± 0.10), it is possible to conclude that the introduction of the two units of the short CCK2R-binding peptide motif leads, as expected, to an increase in overall lipophilicity. Binding to serum proteins was also similar for both compounds, with relatively high (>50%) and overall consistent levels over time. The values observed were in agreement with the ones of the previously published [^68^Ga]Ga-SulfoCy7-FSC-MG and distinctively higher than the ones reported for [^68^Ga]Ga-SulfoCy7-DAFC (22.6 ± 1.8%, 2 h after incubation), again indicating the distinct influence of the targeting peptides in this property [20,32]. The cell assays confirmed a high CCK2R affinity in the subnanomolar range of the new conjugates and an efficient receptor-mediated uptake of both radiotracers examined. Interestingly, a two times higher uptake of radioactivity was observed for [^68^Ga]Ga-CyFMG when compared to [^68^Ga]Ga-CyTMG in the head-to-head cell uptake studies. The cell uptake values observed for the newly developed bimodal imaging agents, [^68^Ga]Ga-CyTMG and [^68^Ga]Ga-CyFMG, were about two and five times higher than the value of 4.5% reported for [^68^Ga]Ga-SulfoCy7-FSC-MG [20]. This improvement could be explained by the optimized amino acid sequence used [33]. The high specific and receptor-mediated internalization to a cytoplasmic location was confirmed by fluorescence microscopy. Of note, heterogeneous uptake of the compounds by the CCK2R-expressing cells was observed in the microscopy images. However, this was found to be consistent with findings from CCK2R-targeting optical imaging agents such as QE, bivQ, and dQ-MG-754 [18,19].

Preliminary in vivo studies of [^68^Ga]Ga-CyTMG in healthy mice demonstrated the expected stability improvement using the stabilized MGS5 sequence for targeting CCK2 receptors (Figure S23). This was further substantiated by the specific in vivo accumulation of both investigated compounds in the CCK2R tumour xenografts. In particular, at 2 h p.i., the ratio between A431-CCK2R and A431-mock tumours of 3.94 achieved for [^68^Ga]Ga-CyFMG was found to be higher in comparison to the value of 3.01 for the [^68^Ga]Ga-SulfoCy7-FSC-MG previously studied [20]. In addition, significant improvements in the tumour-to-organ ratios were observed in the case of the spleen, liver, muscles, and femur. In our perspective, the higher targeting properties and background contrast were mainly achieved by the optimized and metabolically stabilized peptide sequence considered.

The in vivo fluorescence imaging study up to 72 h p.i. indicated that the pharmacokinetic profile of [^68^Ga]Ga-CyFMG in terms of target-to-background contrast is comparable to that of [^68^Ga]Ga-SulfoCy7-FSC-MG. A higher accumulation and prolonged retention in the kidneys was observed for [^68^Ga]Ga-CyTMG instead, indicating a distinctive influence of the chelator on the pharmacokinetic behaviour at later timepoints, resulting in the observed renal washout. Nevertheless, the retention in the kidneys does not represent a significant limitation in terms of radiation dose. This is evidenced by the recent development of [^68^Ga]Ga-Trivehexin [30], which exhibits comparable renal uptake in preclinical models. In no clinical scenario would this off-target accumulation pose any risk of interfering with the detection of kidney metastases. Patients with disseminated CCK2R-positive tumours, like MTC or SCLC, would simply not undergo surgical treatment.

## 4. Materials and Methods

A detailed synthetic procedure for the preparation of CyTMG and CyFMG, analytical data, and a complete list of reagents is provided in the Supplementary Materials.

### 4.1. Instrumentation

#### 4.1.1. Analytical [Radio]-RP-HPLC

RP-HPLC analysis was performed on an UltiMate 3000 system equipped with a pump, autosampler, column compartment, diode array detector (Thermo Fisher Scientific, Vienna, Austria), and radio detector (GabiStar, Raytest; Straubenhardt, Germany).

#### 4.1.2. MALDI-TOF MS

Matrix-assisted laser desorption/ionization time-of-flight mass spectrometry was performed on a Bruker microflex benchtop MALDI-TOF MS (Bruker Daltonics, Bremen, Germany) with reflector acquisition mode and positive ion source settings. Samples were prepared by the dried droplet method using the α-cyano-4-hydroxy-cinnamic acid (HCCA) matrix and the Micro Scout target (MSP96 target ground steel BC, Bruker Daltonics). The recorded data were analyzed using Flex Analysis 2.4 software.

#### 4.1.3. ^68^Ge/^68^Ga Generator

^68^GaCl_3_ was obtained from a commercial ^68^Ge/^68^Ga generator (Eckert and Ziegler, Berlin, Germany) eluted with 0.1 N HCl solution (Rotem Industries, Dimona, Israel). The fractionated elution method was used to increase the ratio of activity to volume to its maximum (150–200 MBq in 1.5 mL).

#### 4.1.4. γ- Counter

The 2480 Automatic Gamma counter Wizard2 3″ (PerkinElmer Life Sciences and Analytical Instruments, formerly Wallac Oy, Turku, Finland) was used to measure the radioactivity of the samples.

#### 4.1.5. Radio-iTLC

Radio instant thin-layer chromatography (radio-ITLC) analysis was performed using iTLC-SG stripes (Agilent Technologies, Folsom, CA, USA) and 0.1 M sodium citrate solution (pH 5). The strips spotted with samples were analyzed using a TLC scanner (Scan-RAM, LabLogistic, Sheffield, UK). Gallium-68 labelling bioconjugates remained at the origin (Rf < 0.1), while the unbound radionuclide migrated to the solvent front (Rf > 0.9).

#### 4.1.6. Confocal Imaging

Real-time live confocal imaging was performed with a spinning disc confocal system (UltraVIEW VoX; Perkin Elmer, Waltham, MA, USA) connected to a Zeiss AxioObserver Z1 microscope (Zeiss, Oberkochen, Germany) equipped with a 40× water immersion objective. DNA staining was performed with HOECHST 33342 (Thermo Fisher Scientific, Vienna, Austria), while cell membrane staining was obtained by using wheat germ agglutinin (WGA-Alexa Fluor 488; Thermo Fisher Scientific, Vienna, Austria).

#### 4.1.7. Cryo-Fluorescence Tomography (CFT)

Cryo-fluorescence tomography was performed using a cryomacrotome (Xerra^TM^, Emit Imaging, Baltimore, MD, USA) and Xerra Controller software v.2.2.4.0 (Emit Imaging). Raw white light and fluorescent images were reconstructed in Xerra Reconstruction software (Emit Imaging) and MHD files (MetaImage Metaheader files) were generated for each subject for white light and fluorescent images.

### 4.2. In Vitro Methods

#### 4.2.1. Radiolabelling with Gallium-68/Radiochemistry

For CyTMG, 2 nmol of the labelling precursor was incubated with 100–200 µL eluate (12.5–25 MBq) and with 10–20 µL of 1.1 M sodium acetate solution to obtain a final pH of 3. The same conditions were used for CyFMG; however, in this case, 20–42 µL of sodium acetate solution was used to achieve a pH of 4.5. ^68^Ga labelling was accomplished within 10 min at 95 °C for [^68^Ga]Ga-CyTMG and at RT for [^68^Ga]Ga-CyFMG. The purity of the radiolabelled compounds was determined both by radio-RP-HPLC with method A(G) (see Supplementary Materials) and by radio-iTLC.

#### 4.2.2. Distribution Coefficient (*LogD*), Stability in Human Serum and PBS, and Protein Binding

For lipophilicity determination, [^68^Ga]Ga-CyTMG and [^68^Ga]Ga-CyFMG labelling solutions were diluted with phosphate-buffered saline (PBS, pH7.4) to a concentration of 0.1 µM. A total of 300 µL of this solution was added to an Eppendorf tube containing 300 µL of octan-1-ol. The mixture was shaken at 1400 rpm and at RT for 15 min and centrifuged at 4500× *g* rpm for 2 min for phase separation. Afterwards, 100 µL of the organic phase and of the aqueous phase were taken out and the activities of the aliquots were quantified in a γ-counter (n = 6). The *LogD* values were calculated with the following formula:

(1) $${LogD}_{pH7.4}=\log_{10\;}\;\left(\frac{{CPM}_{octanol\;phase}}{{CPM}_{\;PBS\;phase}}\right)$$

For stability and protein binding studies, the labelling solutions were diluted with PBS or human serum to a concentration of 0.5 µM and incubated at 37 °C for 1 h, 2 h, and 4 h (n = 3). In the case of serum stability for each timepoint, 100 uL of the incubated solution was diluted 1:1 with ACN to make the proteins precipitate and the solution was centrifuged for 2 min at 14,000× *g* rpm. A total of 100 uL of the supernatant was then diluted 1:1 with water and analyzed by radio-HPLC. For stability in PBS, 100 uL of the incubated solution was diluted 1:1 with water and analyzed by radio-HPLC for every timepoint.

For protein binding determination, MicroSpin G-50 columns (Sephadex G-50, GE Healthcare, Vienna, Austria) were used to separate the fraction of the radio compound bound to human serum proteins from the unbound fraction (n = 2). Before use, storage buffer was removed by centrifugation for 2 min at 2000× *g* rcf. For each timepoint, the column was loaded with 25 uL of the incubated solution and centrifuged for 2 min at 2000× *g* rcf. The resulting activities in the column and in the eluate were measured in the γ-counter. Protein binding was determined with the following formula:

(2) $$Protein\;bound\;fration\;(\%)=\left[\frac{{CPM}_{eluate}}{{(CPM}_{column}+{CPM}_{eluate})}\right]\times 100$$

#### 4.2.3. Tumour Cell Lines and Cell Culture

The human squamous carcinoma cell line A431 transfected with human CCK2R (A431-CCK2R) and with the empty vector alone (A431-mock) were originally provided by Dr. Luigi Aloj (Nuclear Medicine Unit, Istituto Nazionale per lo Studio e la Cura dei Tumori “Fondazione Giovanni Pascale”—IRCCS, Naples, Italy) [34]. All cell culture media and reagents were purchased from Gibco, Invitrogen (Thermo Fisher Scientific, Vienna, Austria), or Sigma-Aldrich (Darmstadt, Germany) Corporation. Dulbecco’s Modified Eagle’s medium (DMEM) supplemented with 10% (*v*/*v*) fetal bovine serum, FBS (10270, Invitrogen, Thermo Fisher Scientific, Waltham, MA, USA), and 1% (*v*/*v*) penicillin–streptomycin–glutamine, PSG (10378, Gibco, Thermo Fisher Scientific, Waltham, MA, USA), was used for cell culture. The cells were grown at 37 °C in a humidified atmosphere with 5% carbon dioxide and were passaged 3 times per week using a 2.5% trypsin–EDTA solution.

#### 4.2.4. Cell Uptake Studies

The cell internalization of the radiolabelled compounds was measured on A431-CCK2R and A431-mock cells. A total of 1.0 × 10^6^ cells per well were seeded in 6-well culture plates (Greiner Cellstar, Sigma-Aldrich Handels GmbH, Vienna, Austria) and grown for 48 h. On the day of the experiment, each well was washed twice with internalization medium (culturing medium supplemented with 1% (*v*/*v*) FBS) and then incubated with 0.5 nM of a radioactive compound for 1 h at 37 °C. After the incubation, the medium was removed and the cells rinsed with 2 × 1 mL of PBS/0.5% (*w*/*v*) bovine serum albumin (BSA). Thereafter, they were washed twice with 1 mL of 50 mM glycine buffer (pH 2.8) with 0.1 M NaCl to remove the membrane-bound radiocompound. Finally, the cells were lysed with 2 × 1 mL of 1 M NaOH to determine the internalized radioligand. All fractions were measured in the γ-counter and the percentage of internalized and membrane-bound radiocompounds in relation to the total radioactivity added to the cells was reported.

#### 4.2.5. Fluorescence Microscopy Studies

Fluorescent tracer uptake was analyzed on A431-CCK2R and A431-mock cells. A total of 3.0 × 10^4^ cells per well were seeded in µ-Slide 8-well plates (Ibidi GmbH, Gräfelfing, Germany) 48 h prior the experiment. The cells were washed with fresh medium and then incubated with metal-free CyTMG or CyFMG (50 nM final concentration) for 30 min. After washing, HOECHST and WGA-Alexa Fluor (final concentration of 5 µg/mL) were added 5 min before microscopy for nuclei and cell membrane staining, respectively. Images were acquired with the same microscope settings (λ_exc_ = 405 nm for HOECHST, λ_exc_ = 488 nm for WGA-Alexa Fluor, and λ_exc_ = 688 nm for the fluorescent tracers).

#### 4.2.6. Receptor-Binding Affinity Studies

The CCK2 receptor-binding affinity of the metal-free CyTMG, CyFMG, and the pentagastrin used as reference, was evaluated in competition assays against [^177^Lu]Lu-DOTA-MGS5 on A431-CCK2R cells. A total of 5 µg of DOTA-MGS5 were radiolabelled with 20 µL of [^177^Lu]LuCl_3_ solution (300–400 MBq; 90–95 GBq/µmol) and 30 µL of 0.4 M sodium acetate/0.24 M gentisic acid solution with pH 5. The labelling mixture was heated at 90 °C for 20 min. At the end, the solution was diluted to a concentration of 200 nM and stored in a freezer for further use. Binding assays were carried out in 96-well filter plates (Multi-ScreenHTS-FB, Merck Group, Darmstadt, Germany) pretreated with 10 mM TRIS/139 mM NaCl buffer at pH 7.4 (TRIS-buffer) (2 × 250 μL) before 400,000 A431-CCK2R cells per well were added in 20 mM HEPES buffer at pH 7.4 containing 10 mM MgCl_2_, 14 μM bacitracin, and 0.5% (*w*/*v*) bovine serum albumin (BSA) (binding assay buffer). Competition assays were performed two times in triplicate using increasing concentrations of competitors (0.001–1,000 nM) and a constant amount of radioligands (~30,000 cpm, 0.3 nM). After 1 h of incubation at room temperature (RT), the medium was removed by vacuum filtration and the filters were rapidly rinsed with ice-cold binding assay buffer (2 × 200 μL), removed, and counted in the γ-counter. Half-maximal inhibitory concentration (IC_50_) values were calculated following non-linear regression with Origin software (MicroCal Origin 6.1, Northampton, MA, USA). For graphical presentation, one exemplary data point set for each compound was normalized to its maximum value.

### 4.3. Animal Experiments

All animal experiments were performed in accordance with the regulations and guidelines of the Czech Animal Protection Act (no. 246/1992) and with the approval of the Czech Ministry of Education, Youth, and Sports (MSMT-24421/2021-4 and MSMT-41830/2018-7) and the institutional Animal Welfare Committee of the Faculty of Medicine and Dentistry of Palacky University in Olomouc. The studies were performed using 10–12-week-old BALB/c or athymic BALB/c nude mice (Envigo, Horst, The Netherlands). Retro-orbital tracer injection, small animal imaging, and sacrification by cervical dislocation were carried out under 2% isoflurane anesthesia (FORANE, Abbott Laboratories, Abbott Park, IL, USA).

#### 4.3.1. Ex Vivo Biodistribution Experiments, Small Animal Imaging Studies

For the ex vivo biodistribution study on healthy BALB/c mice, 3 animals per group were injected with 0.14 nmol of radiotracer (1.5 MBq) and then sacrificed after 1, 2, and 4 h. The organs of interest were extracted, weighed, and measured in the γ-counter. Results were expressed as percentages of injected dose per gram tissue (% ID/g).

For the induction of tumour xenografts, 2 × 10^6^ of A431-CCK2R or A431-mock cells in 200 μL appropriate medium and Matrigel (1:1) were subcutaneously injected in the right and left flank of each mouse (athymic BALB/c nude), respectively. The tumours were allowed to grow until they had reached a volume of 0.3 to 0.6 cm^3^. To evaluate ex vivo biodistribution on tumour models, 3 mouse xenografts were injected with 0.05 nmol of radiotracer (1 MBq) and then sacrificed after 2 h. For the comparative imaging study (PET/CT and in vivo fluorescence imaging), 2 xenograft-bearing mice were injected with 0.3 nmol (6 MBq) of radiotracer. Static PET/CT images of the anesthetized animals in prone position were acquired with a Mediso NanoScan PET/CT small-animal imaging system (Mediso Medical Imaging Systems, Budapest, Hungary) at 1, 2, and 3 h p.i. Image reconstruction was performed via Mediso Tera-Tomo 3D PET iterative reconstruction (Mediso Medical Imaging Systems, Budapest, Hungary). The images were visualized, processed, and quantified in Mediso InterView FUSION (Mediso Medical Imaging Systems, Budapest, Hungary). Quantitative analyses were performed on images of 3 mouse xenografts per compound. The images were normalized to injected activity and animal weight. The results were expressed as percentages of injected dose per gram tissue (% ID/g).

Near-infrared in vivo fluorescence imaging was conducted with an in vivo MS FX PRO small-animal imaging system (Bruker Biospin Corporation, Woodbridge, CT, USA) and image analysis was performed with Bruker MI SE software v. 7.1.1.20220 (Bruker Biospin Corporation, Woodbridge, CT, USA). The mice were positioned in the system in supine position and scanned at various time intervals up to 72 h p.i. by using an appropriate filter set (excitation = 650 nm and emission = 700 nm) and identical illumination settings (acquisition time = 30 s, f-stop = 2.8, field of view = 100 mm, and binning = 4 × 4). The fluorescence emission was reported as photons/s/mm^2^.

#### 4.3.2. Cryo-Fluorescence Tomography (CFT) Data Acquisition

Data were acquired as previously reported [35]. A block mould was prepared and frozen subjects were embedded with optimum cutting temperature for coronal sectioning. The block was frozen at −20 °C prior to being transferred to the cutting stage inside the temperature-controlled cryo-chamber. The block was serially sectioned at either 20 μm or 35 μm and the block face was imaged at each sectioning plane in white light (WL) and fluorescence imaging (Cy5.5, ex 685 nm/ em 711 nm).

### 4.4. Statistical and Data Analysis

All the statistical analyses were performed using Microsoft Office 365 Excel software (Microsoft Corporation, Redmond, WA, USA). The significance of 2 mean values was calculated using an unpaired/independent two-tailed Student’s *t*-test. The level of significance was determined by using the *p* value (*: 0.01 < *p* < 0.05; **: 0.001 < *p* < 0.01; ***: *p* < 0.001).

## 5. Conclusions

In this study, we investigated the possibility of improving the slow blood clearance and non-specific accumulation showed by [^68^Ga]Ga-Sulfo-Cy7-FSC-MG, the first example of a CCK2R-targeting dual-modality imaging agent. For this, we considered a stabilized and higher-affinity targeting unit, a more hydrophilic fluorophore, and considered TRAP as an alternative core scaffold and provided for the first time a head-to-head comparison with Fusarinine C (FSC).

The developed [^68^Ga]Ga-CyTMG and [^68^Ga]Ga-CyFMG showed promising in vitro and in vivo targeting properties along with improvements in the off target uptake, especially in the liver. It was also demonstrated for both compounds that other than PET, fluorescence imaging is also possible with one single injection even at later timepoints, validating the feasibility of the dual modality imaging approach in a murine model. However, further probe optimization, especially in terms of the fluorophore, may improve the imaging performance at early timepoints increasing the potential for clinical translation. Lastly, the major difference observed between [^68^Ga]Ga-CyTMG and [^68^Ga]Ga-CyFMG was the different renal accumulation and retention. This shows that the choice of the chelator for such an approach can have a substantial effect on the pharmacokinetic profile of the final compound and, in particular, influence the biodistribution at late timepoints.

## Figures and Tables

**Table 1:** Results of lipophilicity (*LogD*_pH7.4_), protein binding, and stability determination in PBS and humans serum (% of intact radiotracer) for [^68^Ga]Ga-CyTMG and [^68^Ga]Ga-CyFMG.

		[^68^Ga]Ga-CyTMG	[^68^Ga]Ga-CyFMG
Lipophilicity	*LogD*_pH7.4_ ± SD	−1.78 ± 0.16	−1.53 ± 0.10
	1 h	56.68 ± 6.90	61.39 ± 1.34
Protein binding (%)	2 h	62.26 ± 2.74	65.64 ± 6.01
	4 h	64.09 ± 7.12	66.07 ± 1.26
	1 h	97.98 ± 0.20	99.06 ± 1.07
Stability in PBS (% ± SD)	2 h	97.56 ± 0.09	98.57 ± 0.47
	4 h	96.70 ± 1.15	99.3 ± 0.71
	1 h	98.97 ± 0.81	98.21 ± 1.09
Stability in human serum (% ± SD)	2 h	98.04 ± 0.23	96.80 ± 0.53
	4 h	97.51 ± 0.06	96.94 ± 0.94

## Data Availability

The original contributions presented in the study are included in the article/Supplementary Materials, further inquiries can be directed to the corresponding author/s.

## Supplementary Materials

The following supporting information can be downloaded at: https://www.mdpi.com/article/10.3390/ph17121569/s1, Figure S1: Synthesis scheme for CyTMG and CyFMG; Figures S2–S17: HPLC chromatograms and mass spectra of the labelling precursors and of all the intermediates; Figure S18: Radio-RP-HPLC chromatogram of [^68^Ga]Ga-CyTMG; Figure S19: Radio-iTLC scan of [^68^Ga]Ga-CyTMG; Figure S20: Radio-RP-HPLC chromatogram of [^68^Ga]Ga-CyFMG; Figure S21: Radio-iTLC scan of [^68^Ga]Ga-CyFMG; Figure S22: Radio-HPLC chromatograms of [^68^Ga]Ga-CyTMG and [^68^Ga]Ga-CyFMG after 1, 2, and 4h after incubation in human serum; Figure S23: Radio-HPLC chromatograms of mouse serum and urine 30 min after injection with [^68^Ga]Ga-CyTMG solution. Videos of two different views (coronal and transverse) of the CFT scan of the mouse injected with [^68^Ga]Ga-CyTMG 72h p.i. are available in the journal webpage.

## Abbreviations

ACN	Acetonitrile
BSA	Bovine serum albumin
CCK2R	Cholecystokinin-2 receptor
CFT	Cryo-fluorescence tomography
CuAAC	Cu(I)-catalyzed azide–alkyne cycloaddition
DAFC	Diacetylated Fusarinine C
DMEM	Dulbecco’s Modified Eagle’s medium
DOTA	Dodecane tetraacetic acid
EDTA	Ethylenediaminetetraacetic acid
FDA	Food and Drug Administration
FI	Fluorescence imaging
FSC	Fusarinine C
HEPES	4-(2-hydroxyethyl)-1-piperazineethanesulfonic acid
IC50	Half-maximal inhibitory concentration
ID	Injected dose
iTLC	Instant thin-layer chromatography
MALDI-TOF MS	Matrix-assisted laser desorption/ionization time-of-flight mass spectrometry
MG	Minigastrin
MIP	Maximum-intensity projection
MRI	Magnetic resonance imaging
MTC	Medullary thyroid carcinoma
PBS	Phosphate-buffered saline
PET	Positron emission tomography
PSG	Penicillin–streptomycin–glutamine
Rf	Retention factor
ROI	Region of interest
RP-HPLC	Reversed phase high-performance liquid chromatography
RT	Room temperature
SCLC	Small-cell lung cancer
SPECT	Single-photon emission computed tomography
SulfoCy5.5	Sulfo-Cyanine5.5
TRAP	Triazacyclononane-phosphinic acid
TRIS	Tris(hydroxymethyl)aminomethan
USI	Ultrasound imaging
WGA-Alexa	Wheat germ agglutinin–Alexa
