# Supplementary material for: Comparison of Two Chelator Scaffolds as Basis for Cholecystokinin-2 Receptor Targeting Bimodal Imaging Probes
Source: Pharmaceuticals (Basel). 2024 Nov 22;17(12):1569. doi: 10.3390/ph17121569 (PMC11676163; doi:10.3390/ph17121569)
Supplement: Supplementary file 1 [file pharmaceuticals-17-01569-s001.zip › pharmaceuticals-3310817-supplementary-proof back.pdf]

Supplementary Materials

Table of content

Instrumentation 3
Analytical [radio]-RP-HPLC 3
Preparative RP-HPLC 3
Materials and Methods for the synthesis of the precursors 5
Materials 5
Extraction of Fusarinine C (FSC) 5
Peptide synthesis 6
TRAP-Pr derivatisation_TRAP(alkyne)$_3$ 7
[Fe]FSC derivatisation_[Fe]FSC(alkyne)$_3$ 8
SulfoCy5.5-TRAP(alkyne)2 synthesis 9
SulfoCy5.5-[Fe]FSC(alkyne)$_2$ synthesis 10
CyTMG synthesis 11
CyFMG synthesis 12
RadioHPLC and radio-iTLC for [^{68}Ga]Ga-CyTMG and [^{68}Ga]Ga-CyFMG 13
Human serum stability study for [^{68}Ga]Ga-CyTMG and [^{68}Ga]Ga-CyFMG 15
Metabolic stability in vivo for [^{68}Ga]Ga-CyTMG 16

Instrumentation

Analytical [radio]-RP-HPLC

RP-HPLC analysis was performed on UltiMate 3000 system equipped with pump, autosampler, column compartment, diode array detector (Thermo Fisher Scientific, Vienna, Austria) and radio detector (GabiStar, Raytest; Straubenhardt, Germany).

<u>Method A(A)</u>: A Jupiter 4 µm Proteo 90 Å 250 x 4.6 mm (Phenomenex Ltd. Aschaffenburg, Germany) column with flow rate of 1 mL/min and UV detection at 220 nm was used. Acetonitrile (ACN)/H_2O + 0.1% trifluoroacetic acid (TFA) was used as mobile phase with the following multistep gradient: 0.0-3.0 min 10% ACN, 3.0-16.0 min 10-60% ACN, 16.0-18.0 min 60% ACN, 18.0-18.1 min 60-10% ACN, 18.1-22.0 min 10% ACN.

<u>Method A(B)</u>: A Jupiter 4 µm Proteo 90 Å 250 x 4.6 mm (Phenomenex Ltd. Aschaffenburg, Germany) column with flow rate of 1 mL/min and UV detection at 220 nm was used. Acetonitrile (ACN)/H_2O + 0.1% trifluoroacetic acid (TFA) was used as mobile phase with the following multistep gradient: 0.0-3.0 min 20% ACN, 3.0-17.0 min 20-75% ACN, 17.0-18.0 min 75-90% ACN, 18.0-20.0 min 90% ACN, 20.0-21.0 min 90-20% ACN, 21.0-24.0 min 20% ACN.

<u>Method A(C)</u>: A Jupiter 4 µm Proteo 90 Å 250 x 4.6 mm (Phenomenex Ltd. Aschaffenburg, Germany) column with flow rate of 1 mL/min and UV detection at 220 nm was used. Acetonitrile (ACN)/H_2O + 0.1% trifluoroacetic acid (TFA) was used as mobile phase with the following multistep gradient: 0.0-9.0 min 5%-8% ACN, 9.0-14.0 min 8-25% ACN, 14.0-16.0 min 25-60% ACN, 16.0-20.0 min 60% ACN, 20.0-20.1 min 60-5% ACN, 20.1-23.0 min 5% ACN.

<u>Method A(D)</u>: A Jupiter 4 µm Proteo 90 Å 250 x 4.6 mm (Phenomenex Ltd. Aschaffenburg, Germany) column with flow rate of 1 mL/min and UV detection at 220 nm was used. Acetonitrile (ACN)/H_2O + 0.1% trifluoroacetic acid (TFA) was used as mobile phase with the following multistep gradient: 0.0-5.0 min 5% ACN, 5.0-14.0 min 5-35% ACN, 14.0-23.0 min 35-50% ACN, 23.0-23.1 min 5% ACN, 23.1-28.0 min 5% ACN.

<u>Method A(E)</u>: A Jupiter 4 µm Proteo 90 Å 250 x 4.6 mm (Phenomenex Ltd. Aschaffenburg, Germany) column with flow rate of 1 mL/min and UV detection at 220 nm was used. Acetonitrile (ACN)/H_2O + 0.1% trifluoroacetic acid (TFA) was used as mobile phase with the following multistep gradient: 0.0-3.0 min 10% ACN, 3.0-10.0 min 10-50% ACN, 10.0-18.0 min 50-55% ACN, 18.0-18.1 min 55-10% ACN, 18.1-22.0 min 10% ACN.

<u>Method A(F)</u>: A Jupiter 4 µm Proteo 90 Å 250 x 4.6 mm (Phenomenex Ltd. Aschaffenburg, Germany) column with flow rate of 1 mL/min and UV detection at 220 nm was used. Acetonitrile (ACN)/H_2O + 0.1% trifluoroacetic acid (TFA) was used as mobile phase with the following multistep gradient: 0.0-5.0 min 5% ACN, 5.0-16.0 min 5-60% ACN, 16.0-27.0 min 60% ACN, 27.0-27.1 min 60-5% ACN, 27.1-32.0 min 5% ACN.

<u>Method A(G)</u>: A Jupiter 5 µm C18 300 Å 100 x 4.6 mm (Phenomenex Ltd. Aschaffenburg, Germany) column with flow rate of 1 mL/min. Acetonitrile (ACN)/H_2O + 0.1% trifluoroacetic acid (TFA) was used as mobile phase with the following multistep gradient: 0.0-5.0 min 5% ACN, 5.0-16.0 min 5-60% ACN, 16.0-19.0 min 60% ACN, 19.0-19.1 min 60-5% ACN, 19.1 -24.0 min 5% ACN.

Preparative RP-HPLC

RP-HPLC purification was performed on a UltiMate 3000 pump with UltiMate 3000 UV/Vis detector (Thermo Fisher Scientific, Vienna, Austria).

<u>Method P(A)</u>: A Nucleodur 7 µm C18 HTec 250 x 21 mm (Macherey-Nagel, Düren, Germany) column with flow rate of 15 mL/min and UV detection at 440 nm was used. Acetonitrile (ACN)/H_2O + 0.1% trifluoroacetic

acid (TFA) was used as mobile phase with the following multistep gradient: 0.0-5.0 min 3% ACN, 5.0-7.0 min 3-12% ACN, 7.0-17.0 min 12-22% ACN, 17.0-17.5 min 22-3% ACN, 17.5-27.0 min 3% ACN.

Method P(B): A Reprosil-Pur 120-5 μm C18-AQ 250 x 20 mm (Dr.Maisch GmbH, Ammerbuch, Germany) column with flow rate of 8 mL/min and UV detection at 220 nm was used. Acetonitrile (ACN)/H_2O + 0.1% trifluoroacetic acid (TFA) was used as mobile phase with the following multistep gradient: 0.0-8.0 min 7% ACN, 8.0-8.5 min 7-10% ACN, 8.5-25.0 min 10-12% ACN, 25.0-25.5 min 12-70% ACN, 25.5-29.5 min 70% ACN, 29.5-30.0 min 70-7% ACN, 30.0-35.0 min 7% ACN.

Method P(C): A Nucleodur 5 μm C18 HTec 250 x 16 mm (Macherey-Nagel, Düren, Germany) column with flow rate of 8 mL/min and UV detection at 220 nm was used. Acetonitrile (ACN)/H_2O + 0.1% trifluoroacetic acid (TFA) was used as mobile phase with the following multistep gradient: 0.0-8.0 min 10% ACN, 8.0-30.0 min 10-80% ACN, 30.0-34.0 min 80% ACN, 34.0-35.0 min 80-10% ACN, 35.0-40.0 min 10% ACN.

Method P(D): A Reprosil-Pur 120-5 μm C18-AQ 250 x 20 mm (Dr.Maisch GmbH, Ammerbuch, Germany) column with flow rate of 8 mL/min and UV detection at 220 nm was used. Acetonitrile (ACN)/H_2O + 0.1% trifluoroacetic acid (TFA) was used as mobile phase with the following multistep gradient: 0.0-8.0 min 7% ACN, 8.0-8.5 min 7-15% ACN, 8.5-25.0 min 15-40% ACN, 25.0-25.5 min 40-70% ACN, 25.5-29.5 min 70% ACN, 29.5-30.0 min 70-7% ACN, 30.0-35.0 min 7% ACN.

Method P(E): A Eurospher II 100-5 μm C18-A 250 x 8 mm (Knauer, Berlin, Germany) column with flow rate of 2 mL/min and UV detection at 220 nm was used. Acetonitrile (ACN)/H_2O + 0.1% trifluoroacetic acid (TFA) was used as mobile phase with the following multistep gradient: 0.0-5.0 min 10% ACN, 5.0-25.0 min 10-55% ACN, 25.0-26.0 min 55-85% ACN, 26.0-29.0 min 85% ACN, 29.0-30.0 min 85-10% ACN, 30.0-35.0 min 10% ACN.

Method P(F): A Reprosil-Pur 120-5 μm C18-AQ 250 x 20 mm (Dr.Maisch GmbH, Ammerbuch, Germany) column with flow rate of 8 mL/min and UV detection at 220 nm was used. Acetonitrile (ACN)/H_2O + 0.1% trifluoroacetic acid (TFA) was used as mobile phase with the following multistep gradient: 0.0-8.0 min 7% ACN, 8.0-8.5 min 7-15% ACN, 8.5-30.0 min 15-55% ACN, 30.0-30.5 min 55-70% ACN, 30.5-34.5 min 70% ACN, 34.5 -35.0 min 70-7% ACN, 35.0-40.0 min 7% ACN.

Method P(G): A Eurospher II 100-5 μm C18-A 250 x 8 mm (Knauer, Berlin, Germany) column with flow rate of 2 mL/min and UV detection at 220 nm was used. Acetonitrile (ACN)/H_2O + 0.1% trifluoroacetic acid (TFA) was used as mobile phase with the following multistep gradient: 0.0-5.0 min 10% ACN, 5.0-25.0 min 10-70% ACN, 25.0-26.0 min 70-85% ACN, 26.0-29.0 min 85% ACN, 29.0-30.0 min 85-10% ACN, 30.0-35.0 min 10% ACN.

Materials and Methods for the synthesis of the precursors

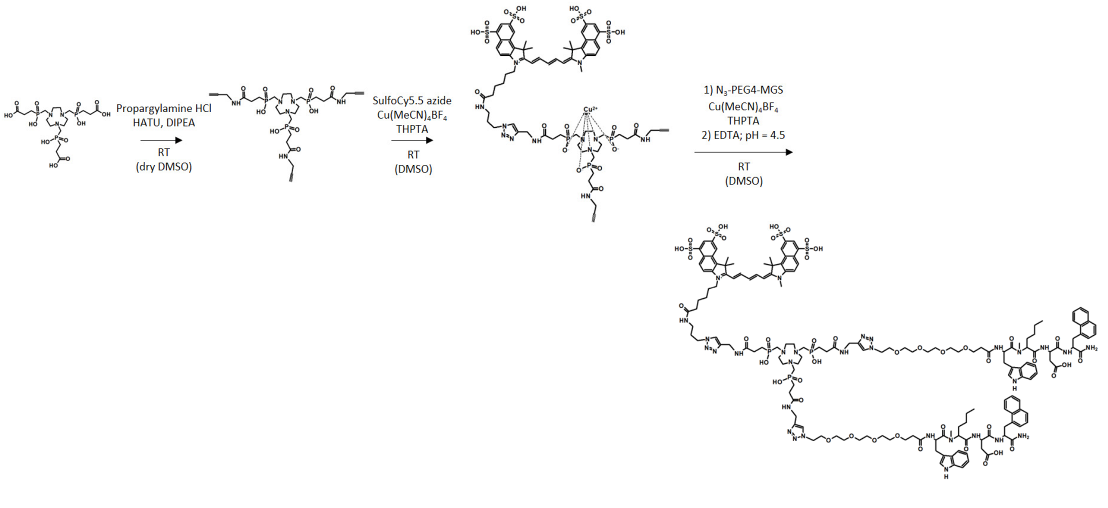

(A)

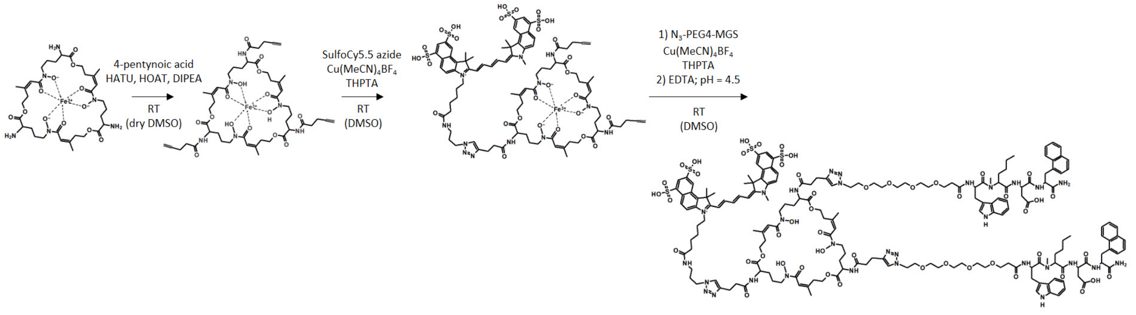

(B)

Figure S1. Synthesis scheme for CyTMG (A) and CyFMG (B) precursors.

Materials

All commercially available chemicals, reagents and solvents were of analytical grade and were used without further purification. Only high-purity water (18 mΩ) was employed. Rink amide MBHA resin (100–200 mesh) and N-terminal 9-fluorenylmethoxycarbonyl (Fmoc) protected amino acids were purchased from Novabiochem (La Jolla,CA,USA). Coupling reagents O-(7-azabenzotriazol-1-yl)- N,N,N´,N´-tetramethyluronium-hexafluorophosphate (HATU) and 1-hydroxy-7-azabenzotriazole (HOAt) were obtained from GenScript Biotech Corporation (Piscataway, NJ, USA). Sulfo-Cyanine5.5 azide and Tris(3-hydroxypropyltriazolylmethyl)amine were purchased from Lumiprobe GmbH (Hannover, Germany) while azido-PEG4-acid was obtained from BroadPharm (San Diego, CA, USA). Trap-Pr was purchased from Fosfinos s.r.o. (Prague, Czech Republich). All other reagents were purchased from Sigma-Aldrich Handels GmbH (Vienna, Austria) and VWR International GmbH (Vienna, Austria).

Extraction of Fusarinine C (FSC)

The siderophore-based natural product fusarinine C (FSC) was obtained from 5L of Aspergillus fumigatus culture prepared under iron deficiency as described by Schrettl and co-workers [1] . After filtering the culture supernatant, $FeSO_4$ or $FeCl_3$ was added in excess to a final concentration of 10 mM obtaining a red

coloured solution. The iron complexation step is necessary to prevent the involvement of the hydroxamates in any side reactions during the synthesis.

The sediment present in the solution was separated by centrifugation (5 min at 6000 rpm and 25°C).

After preparative RP-HPLC purification (gradient P(A); t_R = 14.2 min) and freeze-drying, 735 mg of a red-brown powder was obtained with a purity > 94 % confirmed by analytical RP-HPLC (gradient A(A); t_R = 9.2 min). MS: *m/z* [M+H+] = 780.17 [$C_{33}H_{52}FeN_6O_{12}^+$]; exact mass: 780.30 (calculated)].

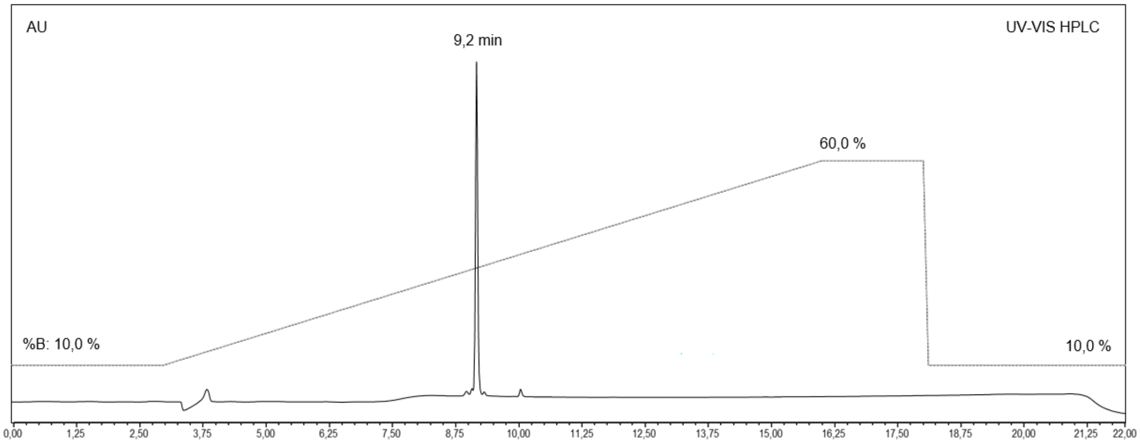

Figure S2. UV-VIS HPLC chromatogram of [Fe]FSC.

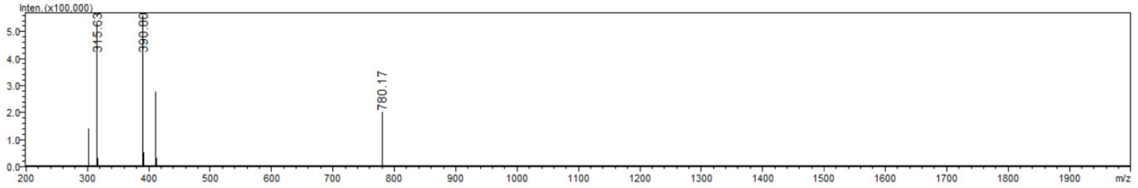

Figure S3. MS spectrum of [Fe]FSC.

Peptide synthesis

N_3-PEG4-MGS [N_3-PEG4-Trp-(N-Me)Nle-Asp-1NaI-NH_2] was prepared by solid-phase peptide synthesis (SPPS) and the coupling efficiency was monitored performing the Kaiser's Test. Fmoc based amino acids were assembled on 300 mg (195 μmol) of 100-200 mesh Novabiochem Rink Amide MBHA resin (Darmstadt, Germany) with 0.65 mmol/g capacity. In order to avoid the reactivity of the carboxyl side chain, the protected amino acids Fmoc-Asp(OtBu)-OH and Fmoc-Trp(Boc)-OH were used.

Before each coupling step, the resin was washed with dimethylformamide (DMF) (6 x with 10 mL for 1 min). The Fmoc protecting groups were cleaved by adding 10 mL of piperidine/DMF (v/v; 20/80) (1x for 5 min, 1x for 15 min) followed by another washing step with dimethylformamide (DMF) (6 x with 10 mL for 1 min). Coupling of amino acids (used in excess) were performed by HATU/HOAt activation in DMF at pH 8-9 and with a reaction time of 45 min.

Before the cleavage, the resin was washed with DMF (5 x with 10 mL for 1 min) and with DCM (2x with 10 mL for 1 min). A solution of TFA/triisopropylsilane/H_2O (v/v; 95/2.5/2.5) was used as a cleavage cocktail (1x with 5 mL for 1h). The cleavage was monitored by RP-HPLC and repeated if necessary. Eventually the solution was diluted with water and concentrated by rotary evaporation.

The purification was performed by preparative RP-HPLC (gradient P(C); t_R = 28.3 min) and yielded 37.5 mg (40.9 µmol, 10.5%) of peptide with a purity > 97% confirmed by analytical RP-HPLC (gradient A(B); t_R = 16.8 min) characterised by MALDI-TOF.

MALDI-TOF-MS: *m/z* [M+Na+] = 938.3, [$C_{46}H_{61}N_9NaO_{11}^+$]; exact mass (monoisotopic): 938.4 (calculated), *m/z* [M+K+] = 954.2, [$C_{46}H_{61}N_9KO_{11}^+$]; exact mass (monoisotopic): 954.4 (calculated)]

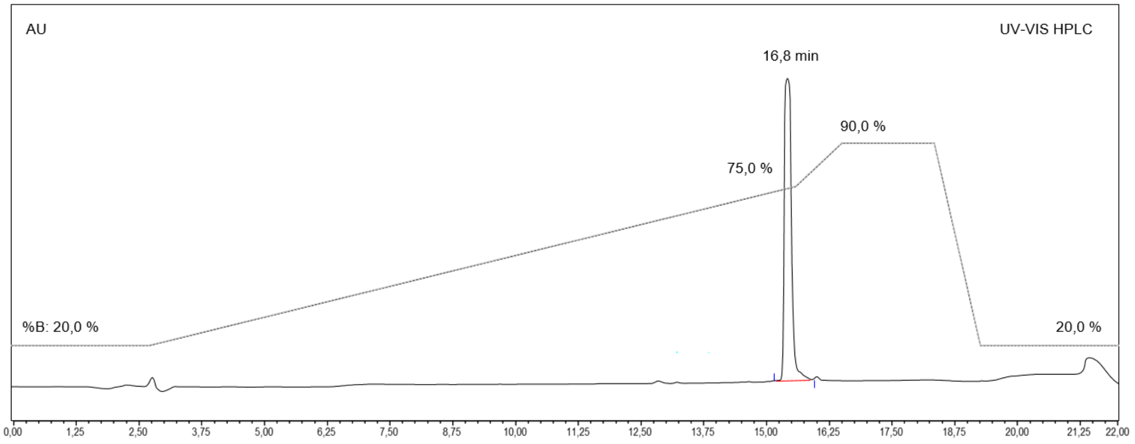

Figure S4. UV-VIS HPLC chromatogram of N_3-PEG4-MGS.

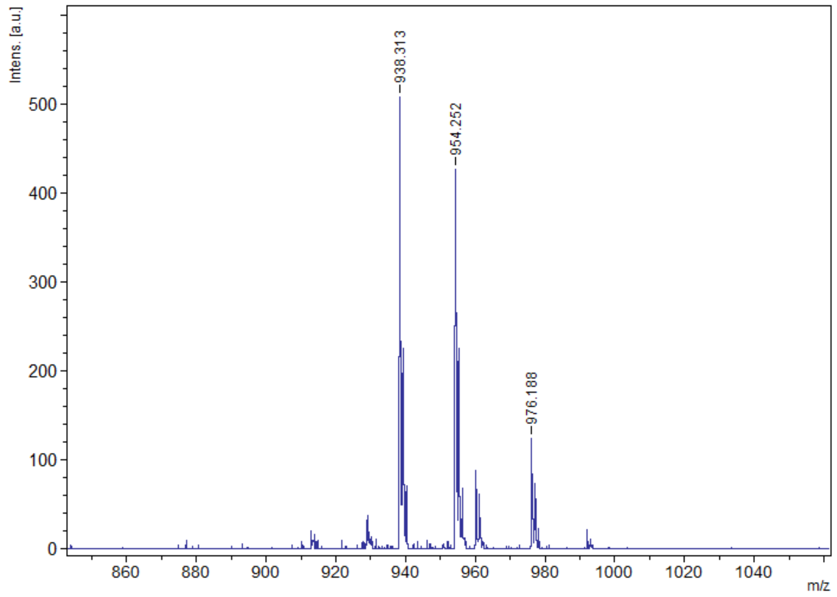

Figure S5. MALDI-TOF-MS spectrum of N_3-PEG4-MGS.

TRAP-Pr derivatisation_TRAP(alkyne)$_3$

TRAP-Pr (100 mg, 162.4 µmol, 1.0 eq.) and propargylamine hydrochloride (74.3 mg, 811.8 µmol, 5 eq.) were dissolved in 0.5 mL of dry DMSO and then 283 µL of DIPEA (1623.7 µmol, 10 eq.) were added. Last, HATU (493.9 mg, 1298.9 µmol, 8 eq.) was added as solid while stirring.

After 1 hour, the solution was dried by rotary evaporation and the residue dissolved in a few millilitres of water.

Preparative RP-HPLC purification (gradient P(B); t_R = 18.9 min) yielded 87.5 mg (126.7 µmol, 78%) of TRAP(alkyne)$_3$ with a purity > 96 % confirmed by analytical RP-HPLC (gradient A(C); t_R = 15.8 min).

MALDI-TOF-MS: *m/z* [M+H+] = 691.2 [$C_{27}H_{46}N_6O_9P_3^+$]; exact mass (monoisotopic): 691.2 (calculated)], *m/z* [M+Na+] = 713.2, [$C_{27}H_{45}N_6NaO_9P_3^+$]; exact mass (monoisotopic): 713.2 (calculated), *m/z* [M+K+] = 729.1, [$C_{27}H_{45}KN_6O_9P_3^+$]; exact mass (monoisotopic): 729.2 (calculated)]

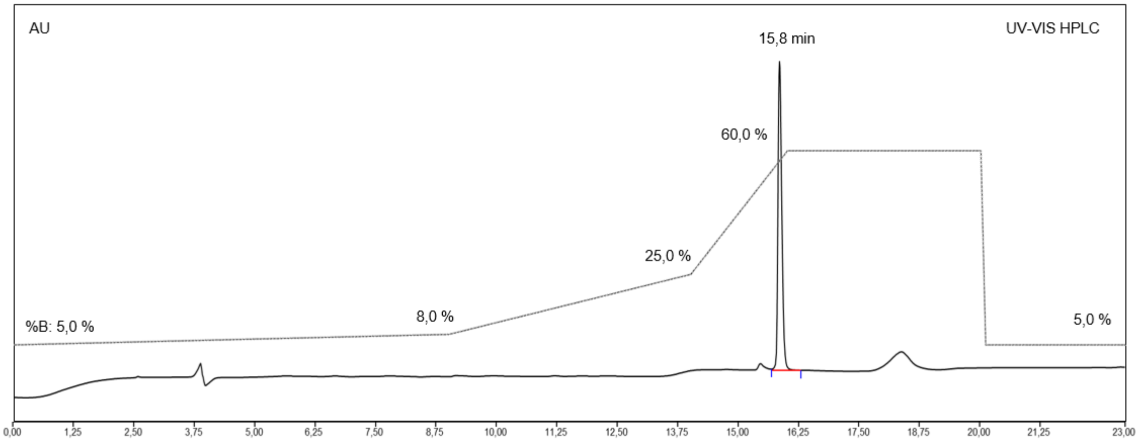

Figure S6. UV-VIS HPLC chromatogram of TRAP(alkyne)$_3$.

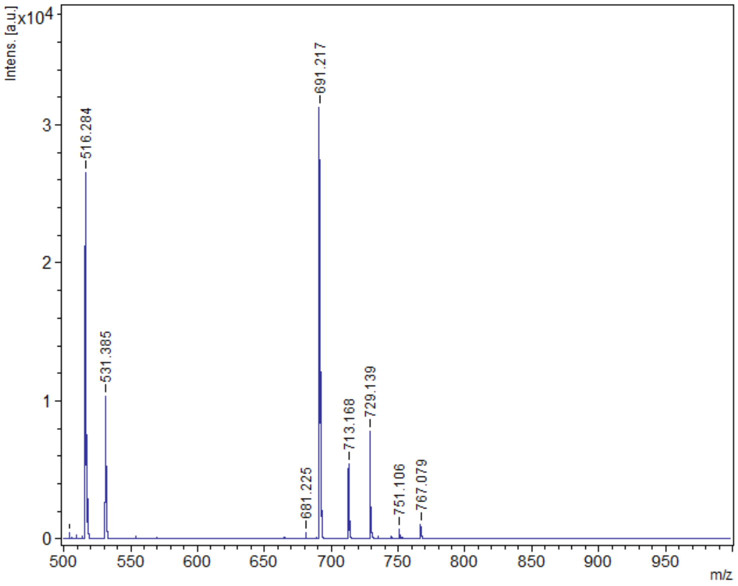

Figure S7. MALDI-TOF-MS spectrum of TRAP(alkyne)$_3$.

[Fe]FSC derivatisation_[Fe]FSC(alkyne)$_3$

60 mg of the siderophore [Fe]FSC (79.6 µmol, 1.0 eq.) were dissolved in 1 mL of dry DMSO and the pH was adjusted to 9 with DIPEA.

365.8 mg of HATU (961.9 µmol) and 37.7 mg of 4-pentynoic acid (384.8 µmol, 5 eq.) were dissolved separately in 1 mL of dry DMSO. The pH was corrected to 9 with 60 µL of DIPEA and the resulting solution was allowed to rest for approximately 5 minutes. This mixture was added dropwise to the solution of the siderophore and the final pH checked and adjusted to 9 if necessary.

After 1 hour, the solution was dried by rotary evaporation and the residue dissolved in a few millilitres of water.

Preparative RP-HPLC purification (gradient P(C); t_R = 27.8 min) yielded 33.4 mg (33.4 µmol, 42%) of [Fe]-FSC(alkyne)$_3$ with a purity > 91 % confirmed by analytical RP-HPLC (gradient A(A); t_R = 16.5 min). MALDI-TOF-MS: *m/z* [M+H$^+$] = 1020.5 [$C_{48}H_{64}FeN_6O_{15}^+$]; exact mass (monoisotopic): 1020.4 (calculated)], *m/z* [M+Na$^+$] = 1042.5, [$C_{48}H_{63}FeN_6NaO_{15}^+$]; exact mass (monoisotopic): 1042.4 (calculated), *m/z* [M+K$^+$] = 1058.4, [$C_{48}H_{63}FeKN_6O_{15}^+$]; exact mass (monoisotopic): 1058.3 (calculated)]

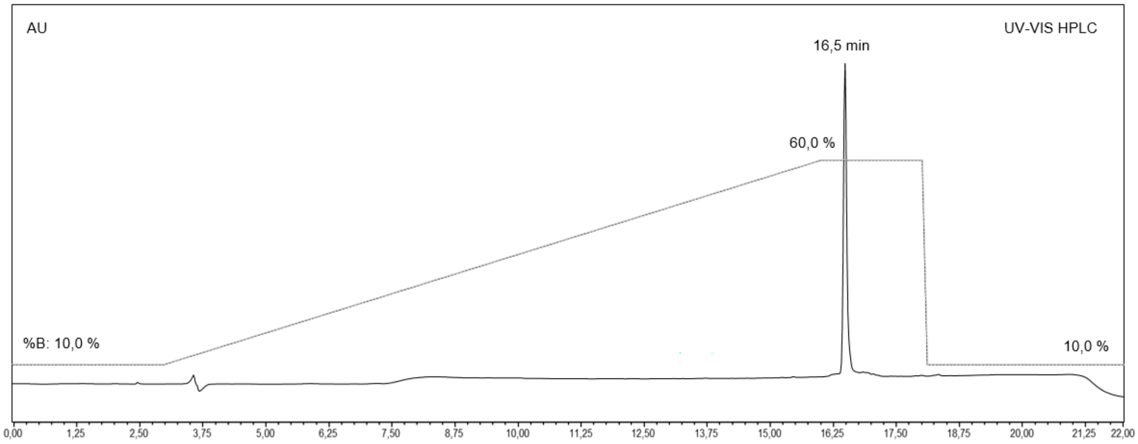

Figure S8. UV-VIS HPLC chromatogram of [Fe]FSC(alkyne)$_3$.

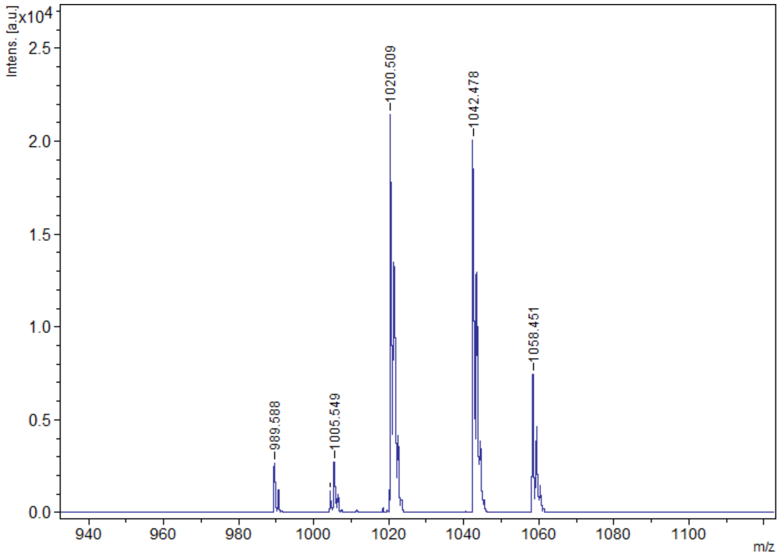

Figure S9. MALDI-TOF-MS spectrum of [Fe]FSC(alkyne)$_3$.

SulfoCy5.5-TRAP(alkyne)$_2$ synthesis

4.5 mg of SulfoCy5.5 azide (4.0 µmol, 1.0 eq.) and 14.1 mg of TRAP(alkyne)$_3$ (20.5 µmol, 5.0 eq.) were dissolved together in DMSO and degassed with Ar flow for 10 min. 9.7 mg of THPTA (22.5 µmol, 5.5 eq) and 7.0 mg of Cu(ACN)$_4$ catalyst (22.5 µmol, 5.5 eq) were dissolved separately in DMSO and degassed with Ar flow for 15 min, then mixed together to give a clear green solution and degassed for further 5 min. Eventually the catalyst solution was added to the solution of azide and alkyne and let react under Ar atmosphere for around 2 hours.

The crude mixture was concentrated by rotary evaporation and purified by preparative RP-HPLC (gradient P(D); t_R = 20 min) yielding 4.1 mg (2.4 µmol, 60%) of SulfoCy5.5-TRAP(alkyne)$_2$ with a purity > 80 % confirmed by analytical RP-HPLC (gradient A(D); t_R = 14.9 min).

MALDI-TOF-MS: *m/z* [M+Cu+] = 1736.4 [$C_{70}H_{92}CuN_{12}O_{22}P_3S_4^+$]; exact mass (monoisotopic): 1736.4 (calculated)].

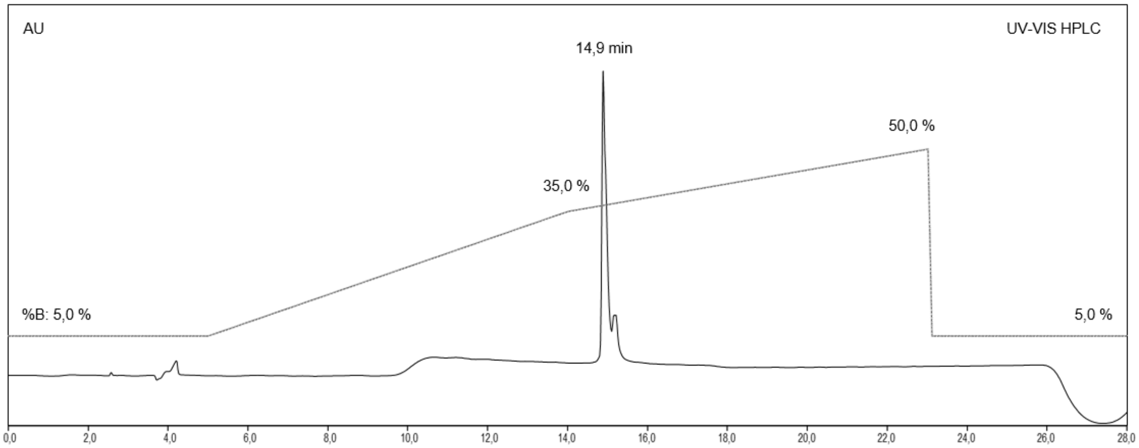

Figure S10. UV-VIS HPLC chromatogram of SulfoCy5.5-TRAP(alkyne)$_2$.

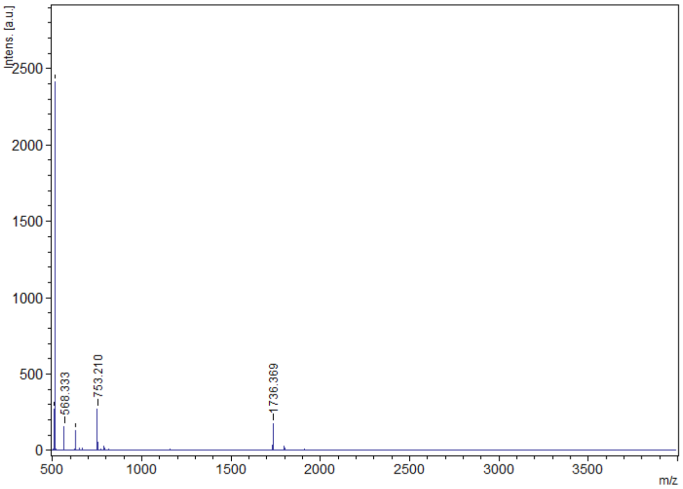

Figure S11. MALDI-TOF-MS spectrum of SulfoCy5.5-TRAP(alkyne)$_2$.

SulfoCy5.5-[Fe]FSC(alkyne)$_2$ synthesis

4.7 mg of SulfoCy5.5 azide (4.3 µmol, 1.0 eq.) and 21.8 mg of [Fe]FSC(alkyne)$_3$ (21.4 µmol, 5.0 eq.) were dissolved together in DMSO and degassed with Ar flow for 10 min. 30.6 mg of THPTA (70.5 µmol, 16.5 eq) and 22.2 mg of Cu(ACN)$_4$ catalyst (70.5 µmol, 16.5 eq) were dissolved separately in DMSO and degassed with Ar flow for 15 min, then mixed together to give a clear green solution and degassed for further 5 min. Eventually the catalyst solution was added to the solution of azide and alkyne and let react under Ar atmosphere for around 2 hours.

The crude mixture was concentrated by rotary evaporation and purified by preparative RP-HPLC (gradient P(E); t_R = 21.1 min) yielding 1.7 mg (0.8 µmol, 18.6%) of SulfoCy5.5-[Fe]FSC(alkyne)$_2$ with a purity > 87 % confirmed by analytical RP-HPLC (gradient A(E); t_R = 11.3 min). FT-MS + pNSI: *m/z* [M+H+]$^{2+}$ = 1002.2 [$C_{91}H_{113}FeN_{12}O_{28}S_4$]$^{2+}$; exact mass (monoisotopic)/2: 1002.8 (calculated)]. *m/z*

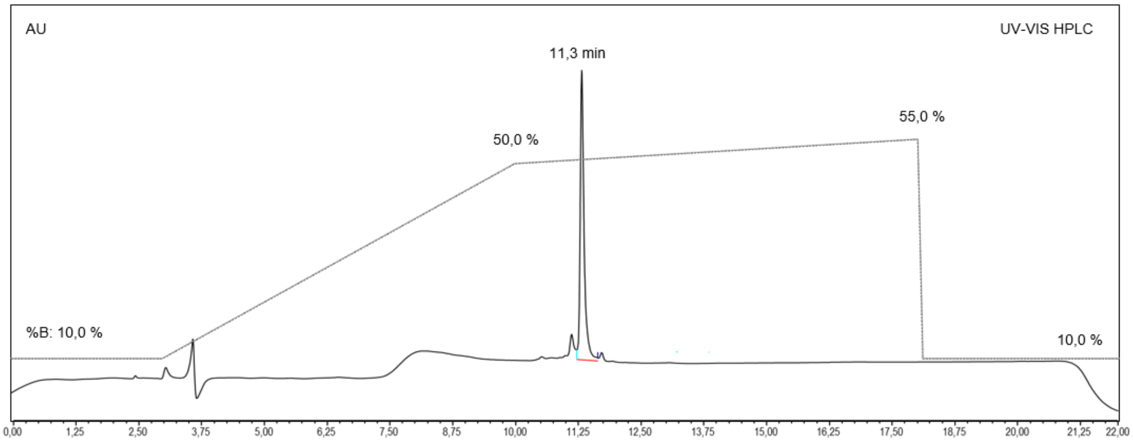

Figure S12. UV-VIS HPLC chromatogram of SulfoCy5.5-[Fe]FSC(alkyne)$_2$.

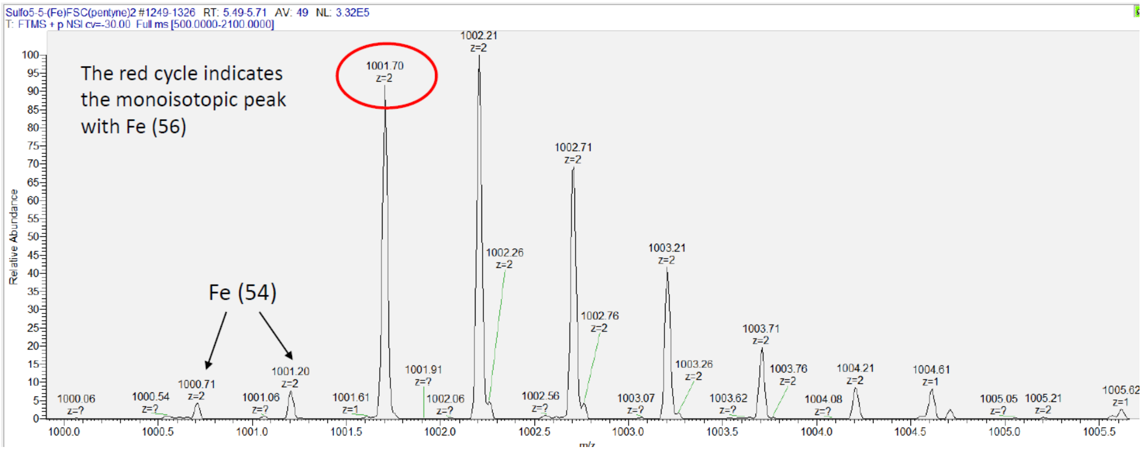

Figure S13. MS spectrum of SulfoCy5.5-[Fe]FSC(alkyne)$_2$.

CyTMG synthesis

3.5 mg of SulfoCy5.5-TRAP(alkyne)$_2$ (2.1 µmol, 1.0 eq.) and 4.6 mg of N_3-PEG4-MGS (5.0 µmol, 2.4 eq.) were dissolved together in DMSO and degassed with Ar flow for 10 min. 7.3 mg of THPTA (16.7 µmol, 8.0 eq) and 5.3 mg of Cu(ACN)$_4$ catalyst (16.7 µmol, 8.0 eq) were dissolved separately in DMSO and degassed with Ar flow for 15 min, then mixed together to give a clear green solution and degassed for further 5 min. Eventually the catalyst solution was added to the solution of azide and alkyne and let react under Ar atmosphere for around 2 hours.

For demetallation, the organic solvent was evaporated. The resulting conjugates were dissolved in 1 mL of a 50% (v/v) H_2O/ACN solvent and an aqueous solution of EDTA (400 mM) was added to provide an excess of approximately 50 eq. of EDTA over the conjugates. The pH was adjusted to 4.5, and the solution was stirred for 4 hours.

The crude mixture was concentrated by rotary evaporation and purified by preparative RP-HPLC (gradient P(F); t_R = 31.5 min) yielding 2.6 mg (0.7 µmol, 33.3%) of CyTMG with a purity > 95 % confirmed by analytical RP-HPLC (gradient A(F); t_R = 17.4 min). FT-MS + pNSI: *m/z* $[M+2H^+]^{3+}$ = 1169.5 $[C_{162}H_{218}N_{30}O_{44}P_3S_4]^{3+}$; exact mass (monoisotopic)/3 = 1169.5 (calculated)].

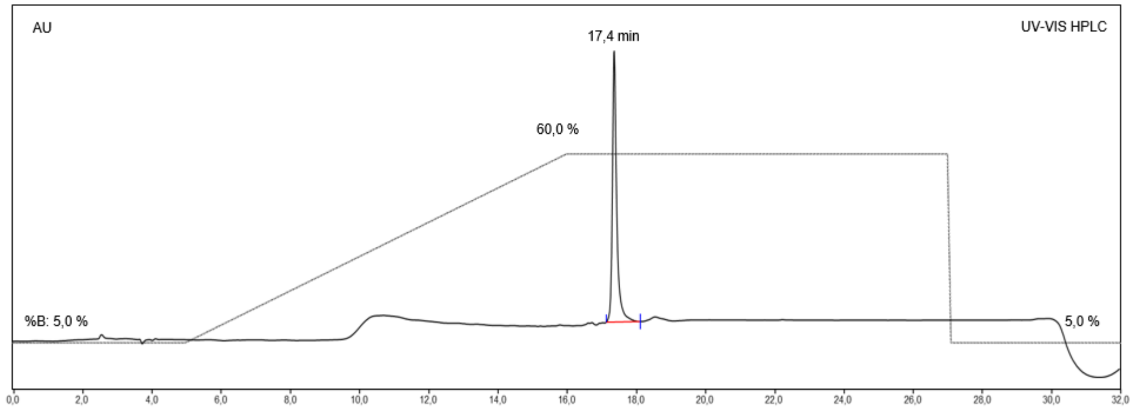

Figure S14. UV-VIS HPLC chromatogram of CyTMG.

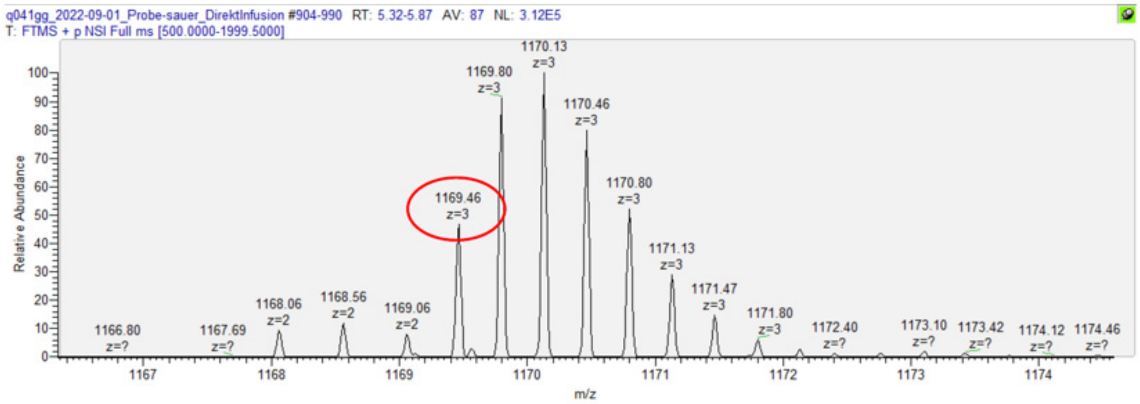

Figure S15. MS spectrum of CyTMG.

CyFMG synthesis

1.7 mg of SulfoCy5.5-[Fe]-FSC(alkyne)$_2$ (0.8 µmol, 1.0 eq.) and 2.3 mg of N$_3$-PEG4-MGS (2.5 µmol, 3 eq.) were dissolved together in DMSO and degassed with Ar flow for 10 min. 3.0 mg of THPTA (6.9 µmol, 8.0 eq) and 2.1 mg of Cu(ACN)$_4$ catalyst (6.9 µmol, 8.0 eq) were dissolved separately in DMSO and degassed with Ar flow for 15 min, then mixed together to give a clear green solution and degassed for further 5 min. Eventually the catalyst solution was added to the solution of azide and alkyne and let react under Ar atmosphere for around 2 hours.

For demetallation, the organic solvent was evaporated. The resulting conjugates were dissolved in 1 mL of a 50% (v/v) H_2O/ACN solvent and an aqueous solution of EDTA (400 mM) was added to provide an excess of approximately 50 eq. of EDTA over the conjugates. The pH was adjusted to 4.5, and the solution was stirred for 4 hours.

The crude mixture was concentrated by rotary evaporation and purified by preparative RP-HPLC (gradient P(G); t_R = 27.4 min) yielding 0.7 mg (186 nmol, 23.2%) of CyFMG with a purity > 95 % confirmed by analytical RP-HPLC (gradient A(G); t_R = 15.5 min). FT-MS + pNSI: *m/z* $[M+2H^+]^{3+}$ = 1261.5 $[C_{183}H_{239}N_{30}O_{50}S_4]^{3+}$; exact mass (monoisotopic)/3: 1261.5 (calculated)].

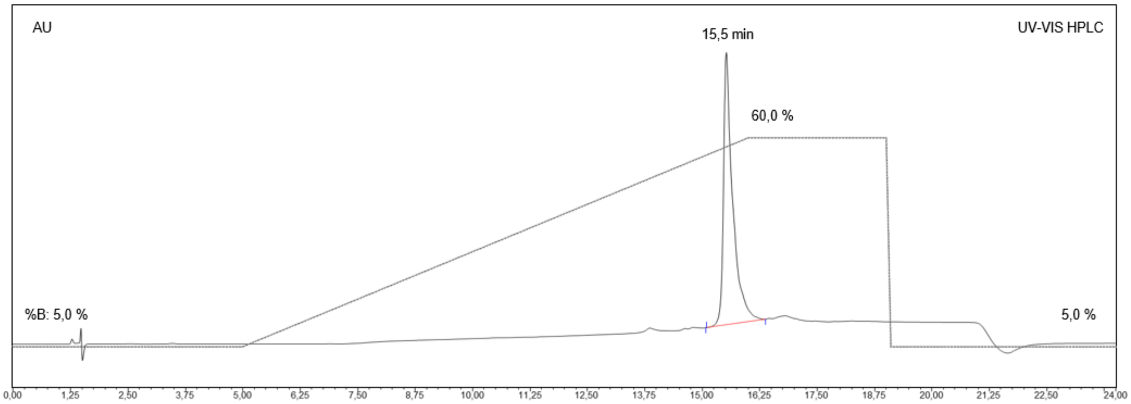

Figure S16. UV-VIS HPLC chromatogram of CyFMG.

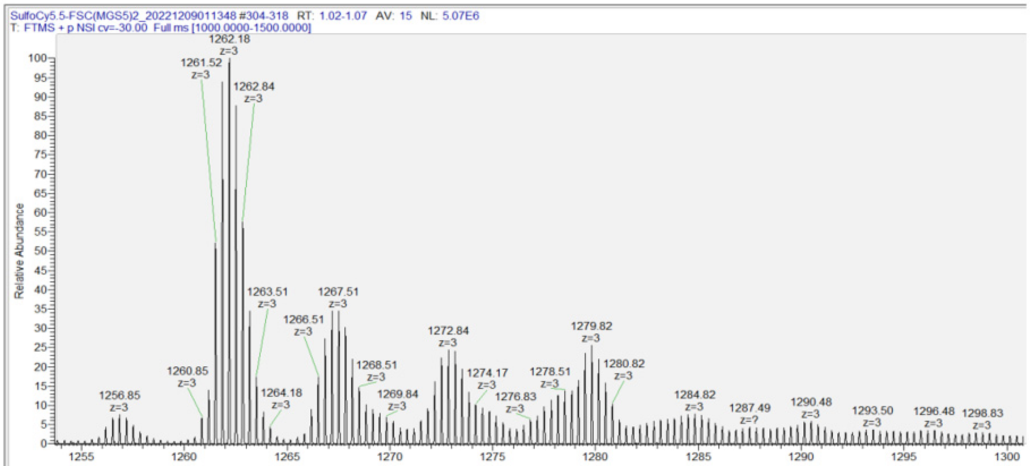

Figure S17. MS spectrum of CyFMG.

RadioHPLC and radio-iTLC for [^{68}Ga]Ga-CyTMG and [^{68}Ga]Ga-CyFMG

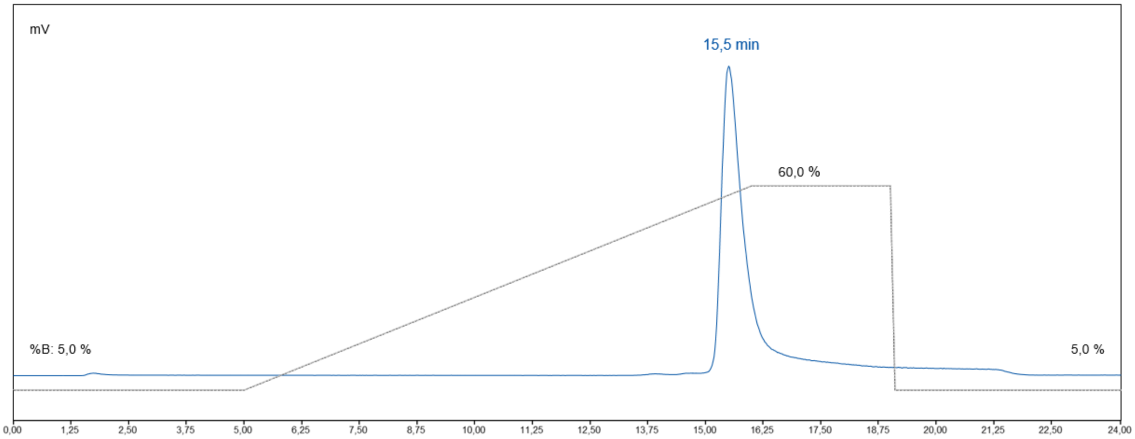

Figure S18. *Radio*-RP-HPLC chromatogram of [^{68}Ga]Ga-CyTMG.

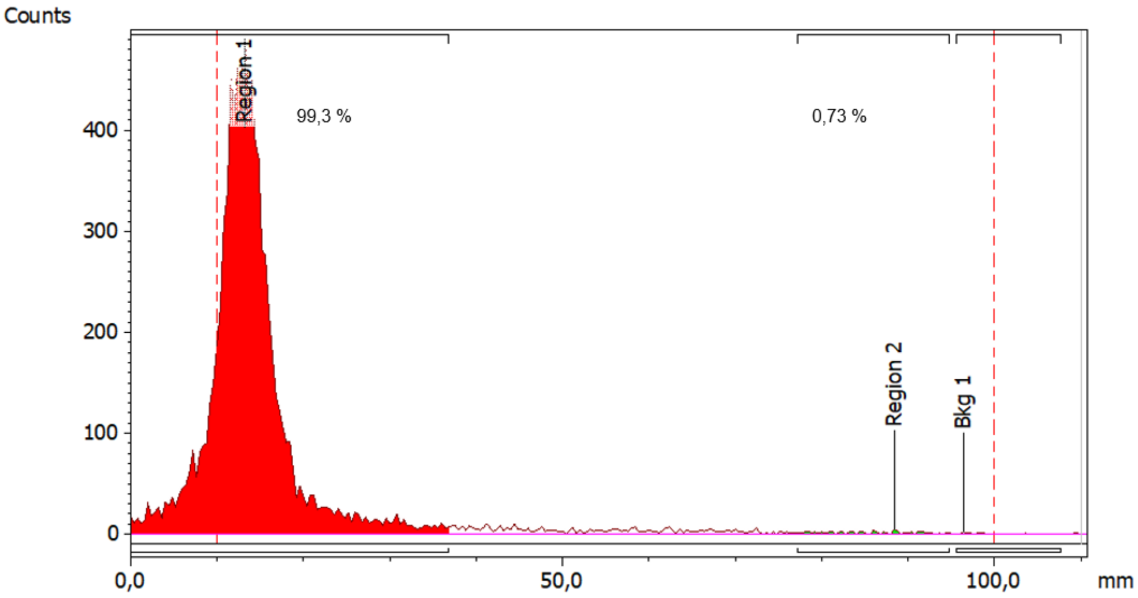

Figure S19. *Radio*-iTLC scan of [^{68}Ga]Ga-CyTMG.

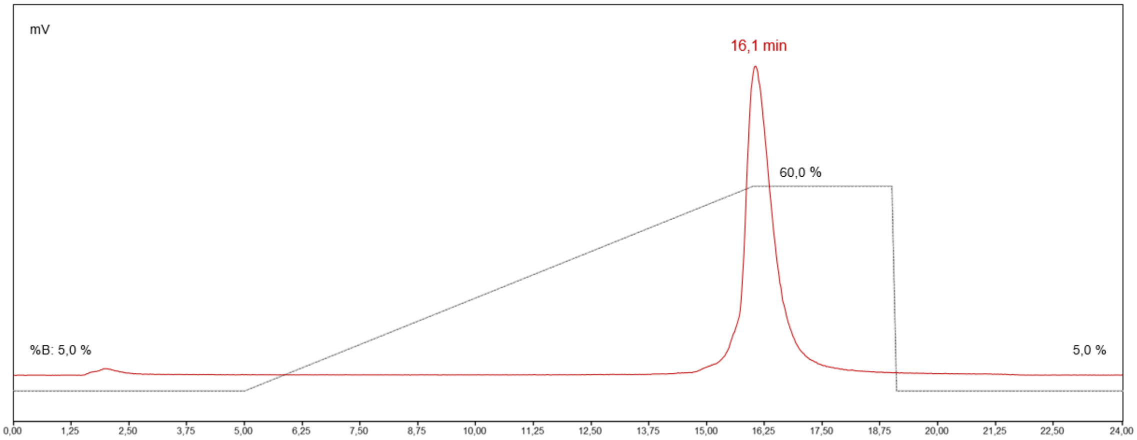

Figure S20. *Radio*-RP-HPLC chromatogram of [^{68}Ga]Ga-CyFMG.

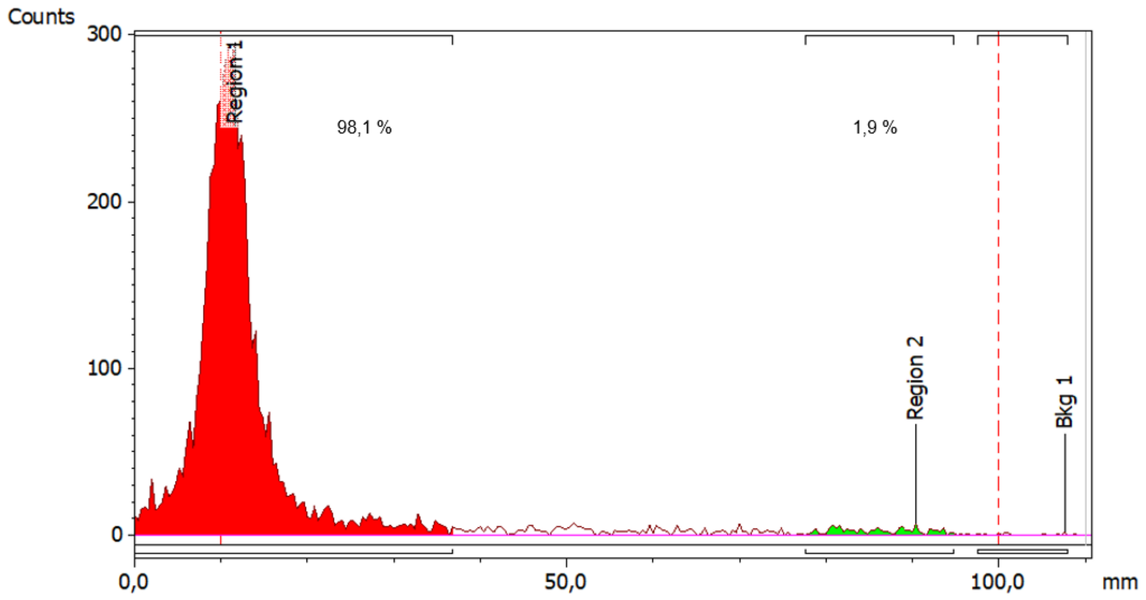

Figure S21. *Radio*-iTLC scan of [^{68}Ga]Ga-CyFMG.

Human serum stability study for [^{68}Ga]Ga-CyTMG and [^{68}Ga]Ga-CyFMG

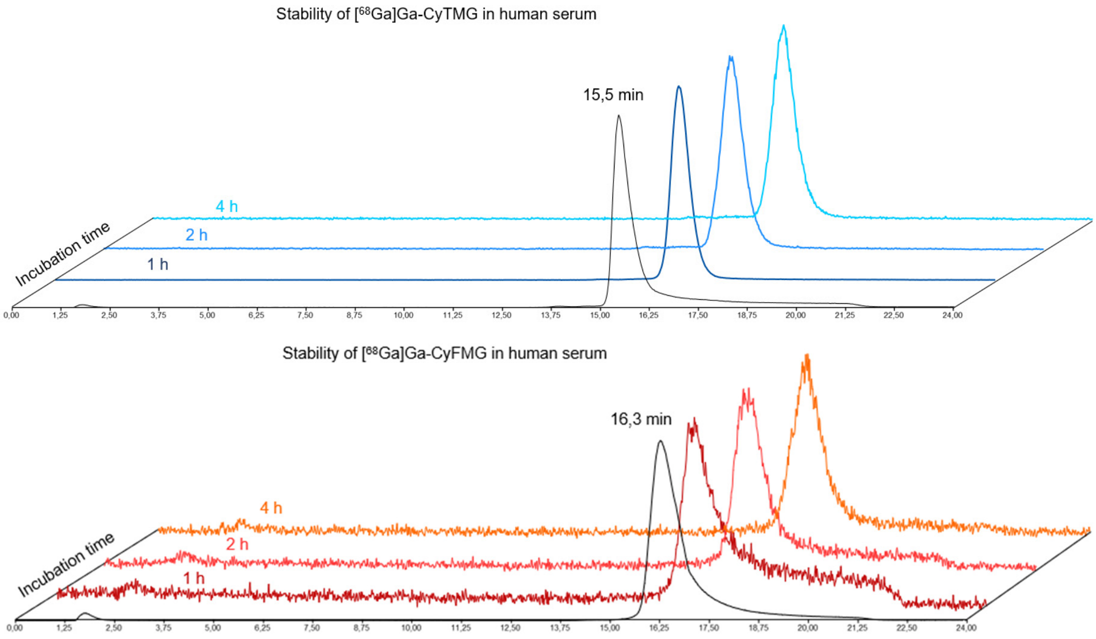

Figure S22. Radio-HPLC chromatograms of [^{68}Ga]Ga-CyTMG and [^{68}Ga]Ga-CyFMG after 1,2 and 4h after incubation in human serum.

Metabolic stability in vivo for [^{68}Ga]Ga-CyTMG

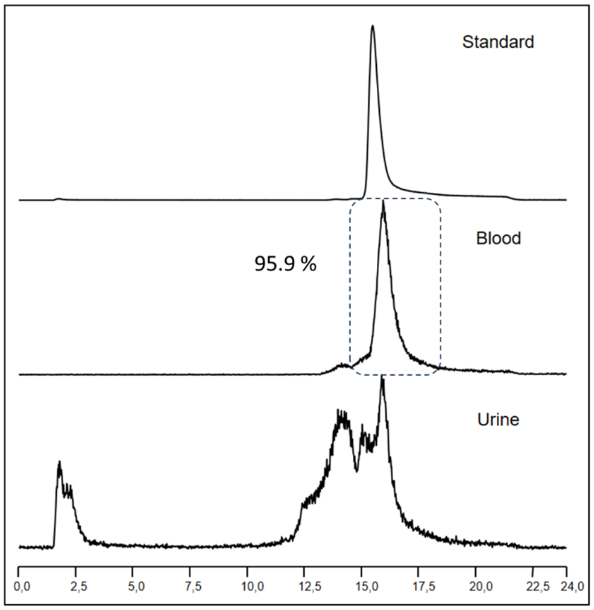

Figure S23. Radio-HPLC chromatograms of mouse serum and urine 30 min after injection with [^{68}Ga]Ga-CyTMG solution (0.75 nmol,17.4 MBq).

One mouse was injected with 0.75 nmol of [^{68}Ga]Ga-CyTMG (17.4 MBq) and sacrificed 30 min p.i. Urine and blood sample were collected at the time of sacrifice. Before radio-RP-HPLC analysis, the blood sample was centrifuged for 2 min at 14000 rpm. 100 µL of the supernatant was diluted and mixed 1:1 with ACN and centrifuged again to separate the protein pellet. An aliquot of the supernatant was diluted 1:1 with water and analysed. Before the analysis the urine sample was solely diluted 1:100 with water.
